# Metal-free synthesis of 1,*N*^6^-ethenoadenines from *N*^6^-propargyl-adenines *via* NIS mediated radical cascade reaction†

**DOI:** 10.1039/c9ra09198j

**Published:** 2019-11-26

**Authors:** Ruchun Yang, Si Deng, Xiang-you Dong, Xianrong Song, Hu Cai, Jiang Bai, Qiang Xiao

**Affiliations:** Institute of Chemistry, Nanchang University Nanchang 330031 Jiangxi Province China caihu@ncu.edu.cn; Institute of Organic Chemistry, Jiangxi Science & Technology Normal University, Key Laboratory of Organic Chemistry Nanchang 330013 Jiangxi Province China xiaoqiang@tsinghua.org.cn

## Abstract

In the present paper, an efficient approach for the construction of 1,*N*^6^-ethenoadenines from conveniently prepared *N*^6^-propargyl-adenines is developed. This reaction merges *N*-iodosuccinimide radical initiation and aerobic aminooxygenation in dioxane. This mild, *5-exo-dig*, and metal-free cascade reaction could be applied to a wide substrate scope to provide 1,*N*^6^-ethenoadenines in moderate to good yields. The reaction mechanism was proposed and tested using radical inhibitor (butylated hydroxytoluene) and isotopic labelling (^18^O_2_) experiments.

## Abstract 2

An efficient approach for the construction of 1,*N*^6^-ethenoadenines is developed through the metal-free mediated radical cascade cyclization of conveniently prepared *N*^6^-propargyl-adenines.

## Graphical abstract

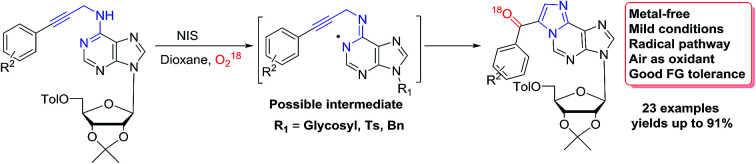

## Introduction

The development of efficient strategies for the synthesis of nitrogen containing heterocycles has attracted tremendous interest from both academic and pharmaceutical companies. In the past decades, various approaches have been developed and great progress has been achieved.^1^ Among these approaches, the cascade reaction turned out to be the most extensively employed one due to its high efficiency and step economy, without the isolation of possible miscellaneous intermediates.^2^ However, the corresponding nitrogen or sulphur atoms in the heterocyclic substrates generally possess strong coordinating ability to metal ions, which could lead to catalyst poisoning and side reactions.^3^ Thus, it is highly desirable to develop a new metal-free cascade reaction particularly for multiple nitrogen-containing heterocycles, such as nucleosides.

**Fig. 1:**
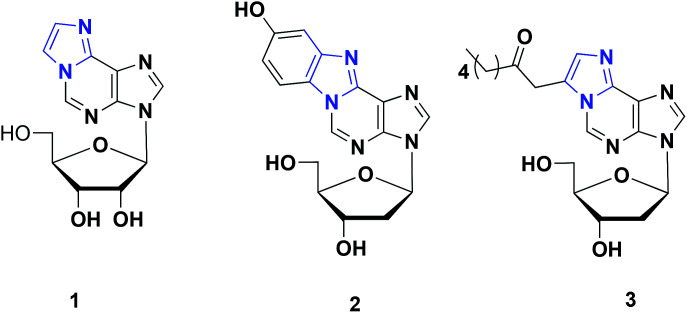
Representative examples of 1,*N*^6^-ethenoadenosines.

In our continuous effort to develop fluorogenic nucleosides for nucleic acid analysis,^4^ 1,*N*^6^-ethenoadenosines have attracted our attention because of their unique biological activities and conjugated structural skeleton (Fig. 1).^5^ Specifically, 1,*N*^6^-ethenoadenine 1 showed strongly fluorescent emission depending on the surrounding environment, which has been extensively used in analyzing the structure and functions of nucleic acids.^6^ Furthermore, it has been also recognized as a biomarker for the study of oxidative stress-related diseases and genetic damages associated with cancer.^7^ In addition, 9-hydroxy-1,*N*^6^-benzetheno-2′-deoxyadenosine 2 was firstly identified as DNA adduct with *p*-benzoquinone formed by peroxidase activation of benzene metabolites, which is well-known to cause acute leukemia in humans and bone marrow toxicity.^8^ Ethenoadenine 3 is another adducts of adenosine with 4-oxo-2-nonenal, which is a novel product of lipid peroxidation and may play an important role in lipid hydroperoxide-mediated carcinogenesis.^9^

Despite of the significance of 1,*N*^6^-ethenoadenosines mentioned above, literatures survey revealed that the availability of their synthetic approaches was very rare. The typical synthetic route is the reaction of α-halocarbonyl compounds with purine.^10^ Therefore, the development of novel methodology with high efficiency and structural diversity is in high demand.

**Scheme 1:**
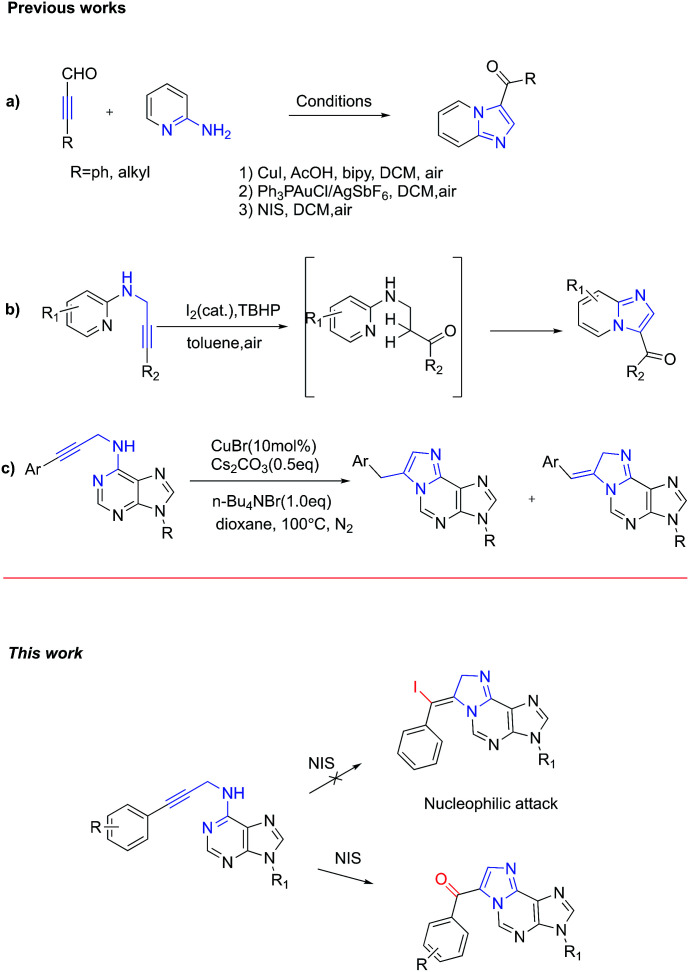
Diverse constructions of imidazo[1,2-*a*]pyridine.

**Scheme 2:**
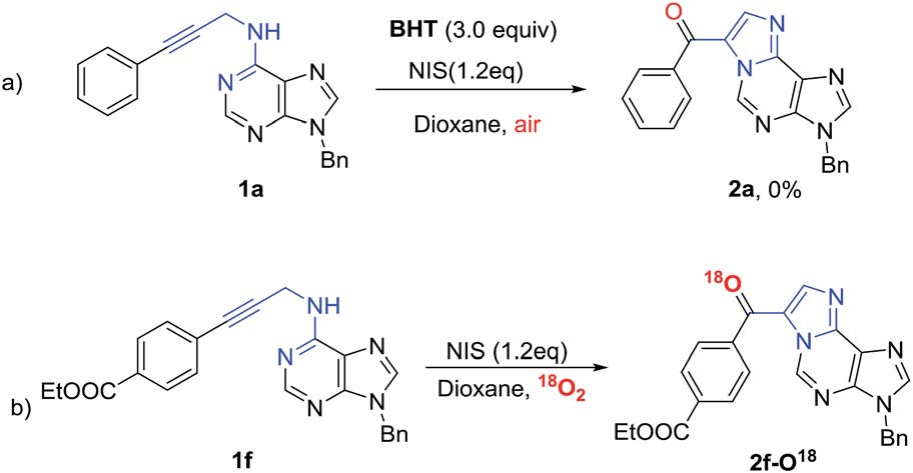
Mechanistic studies and control experiments.

**Scheme 3:**
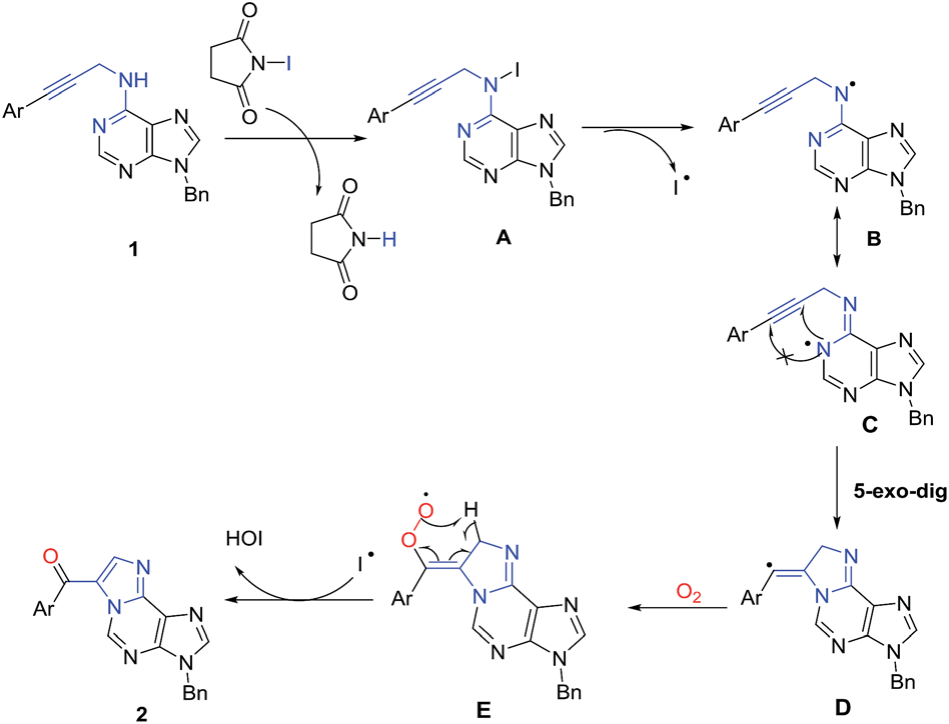
Proposed reaction mechanism.

In recent years, propargylamine derivatives, bearing electrophilic triple bonds, have emerged as promising cascade synthons for heterocycle synthesis.^11^ For example, imidazo[1,2-*a*]pyridines were readily prepared from 3-phenylpropiolaldehyde and 2-aminopyridine by using either copper(i) or gold(i) catalyst along with air as the oxidant (Scheme 1a, (1) and (2)).^12^ Later on, Das *et al.* further developed an improved and general metal-free aminooxygenation of alkynes for the rapid construction of 3-aroylimidazo[1,2-*a*]pyridines (Scheme 1a, (3)).^13^ According to the proposed mechanism, NIS works as an iodine cation donor, which may coordinate with the alkyne to form iodonium intermediate. Then nucleophilic addition of water followed by elimination of hydrogen iodide afforded the target product. Very recently, Huang *et al.* reported a metal free synthesis of aroylimidazo[1,2-*a*]pyridine *via* intramolecular dehydrogenative aminooxygenation of alkynes, which use I_2_ as catalyst and TBHP as an oxidant (Scheme 1b).^14^ But this approach has never been applied to purine substrates. In 2014, Guo *et al.* developed a novel approach to construct purine-fused 1,*N*^6^-ethenoadenine *via* copper-catalysed intramolecular cyclization of *N*^6^-propargyl-adenine at high temperature. However, this approach always affords two regioisomers (Scheme 1c).^15^

Based on examples mentioned previously in the literature, we envisioned that *N*^6^-propargyl-adenine could be activated by NIS to form iodonium intermediate. The subsequent *N*-1 mediated nucleophilic attacking to the resulting iodonium intermediate would generate 1,*N*^6^-ethenoadenines products directly. If it works, this new metal free synthetic route could provide direct access to 1,*N*^6^-ethenoadenines in one step cascade reaction from readily available starting material.

## Results and discussion

To test our hypothesis, we initially mixed readily prepared *N*^6^-propargyl-adenine 1a with 1 eq. NIS in DCM at room temperature (Table 1, entry 1). It is encouraging to find that a florescent product was obtained in moderate yield of 46%. From NMR spectra, there is only one CH_2_ group present which belong to *N*^9^-benzyl group and there is a new carbonyl and a new aromatic CH appeared. In addition, element analysis excluded iodine atom in the target molecule. After extensive characterization analysis, the target structure was determined to be the oxidized cascade cyclization product 2a.

**Table 1:** Optimization of reaction conditions^a^

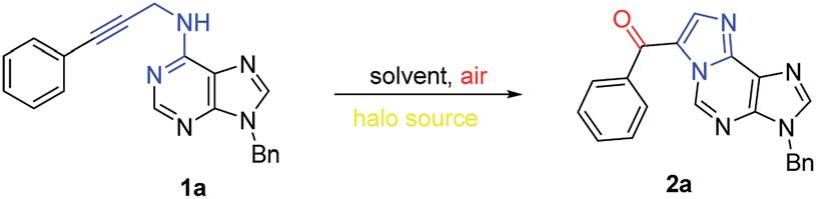				
Entry	Halo source	Solvent	Time	Yield^b^
1	NIS	DCM	12 h	46
2	NIS	CH_3_OH	12 h	34
3	NIS	DMSO	12 h	36
4	NIS	Dioxane	12 h	74
5	NIS	DMF	12 h	23
6	NIS	CH_3_CN	12 h	52
7	NIS	THF	12 h	48
8	—	Dioxane	12 h	None
9	NIS (0.5 eq.)	Dioxane	12 h	33
10	NIS (1.2 eq.)	Dioxane	12 h	75
11	NIS (1.5 eq.)	Dioxane	12 h	73
12^c^	NIS (1.2 eq.)	Dioxane	12 h	65
13^d^	NIS (1.2 eq.)	Dioxane	12 h	63
14	NBS	Dioxane	12 h	Trace
15	NCS	Dioxane	12 h	None
16	I_2_	Dioxane	12 h	13
17^e^	NIS (1.2 eq.)	Dioxane	12 h	46
18^f^	NIS (1.2 eq.)	Dioxane	12 h	Trace

a Reaction conditions: 0.1 mmol 1a, 1.0 eq. NIS, in 2.0 mL dioxane under air, room temperature.

b Isolated yields.

c Reaction performed at 50 °C.

d Reaction performed at 80 °C.

e Dark, 1 atm O_2_.

f Sunlight, argon atmosphere.

In order to improve the efficiency, a series of solvents with different polarity, such as DMF, dioxane, CH_3_CN, CH_3_OH, and DMSO, were screened (entries 1–7). Dioxane proved to be optimal and gave the best yield of 74% (entry 4). Moreover, 1.2 eq. NIS is sufficient to complete this transformation (entry 10). Increasing the reaction temperature led to slightly lower yields (entries 12–13). Furthermore, the effect of different halo sources (NCS, NBS and iodine) were examined (entries 14–16). Neither NBS nor NCS could promote the cascade reaction and only trace amount of desired product could be detected. Using iodine only provided 13% yield. In addition, the reaction performed under argon gave only trace amount of product, revealing the essential role of oxygen. Furthermore, the reaction became sluggish in absence of light. Thus, we chose dioxane as solvent, 1.2 eq. NIS, room temperature, and opening to air as the reaction conditions for further investigation (Table 2).

**Table 2:** Metal-free cascade cyclization–oxidation of *N*^6^-propargyl-adenine^a^

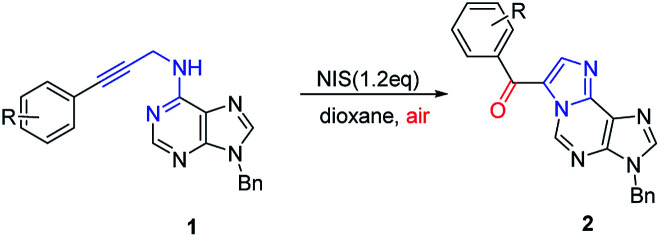
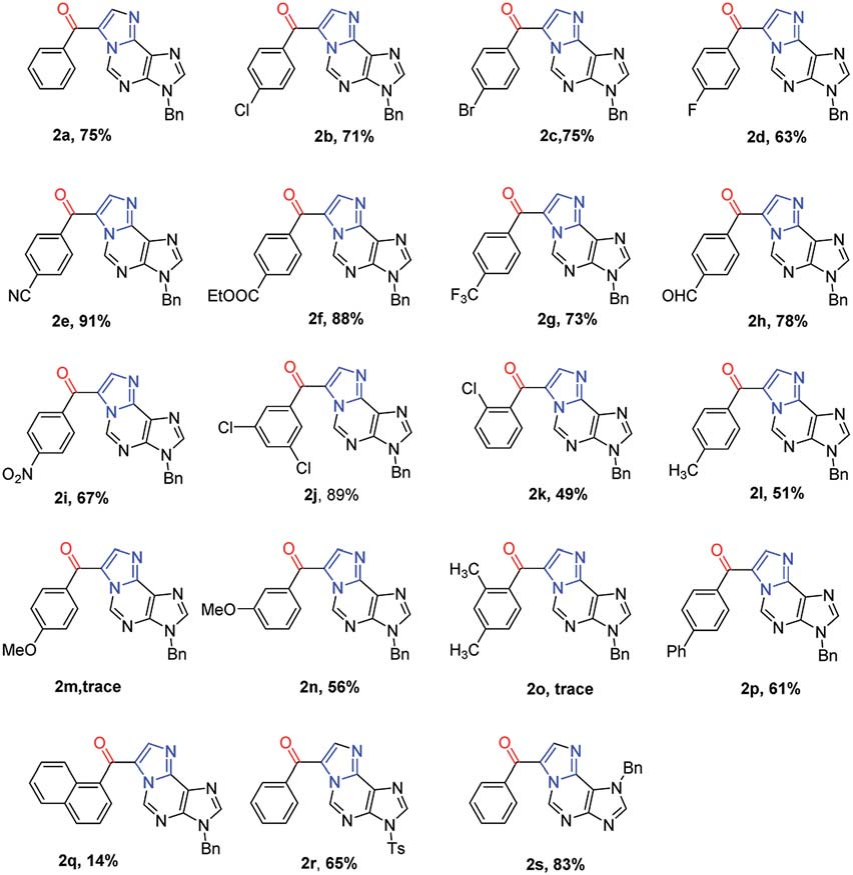

a Reaction conditions: 0.1 mmol 1a–1s, 1.2 eq. NIS, in 2.0 mL dioxane under air; isolated yields.

**Fig. 2:**
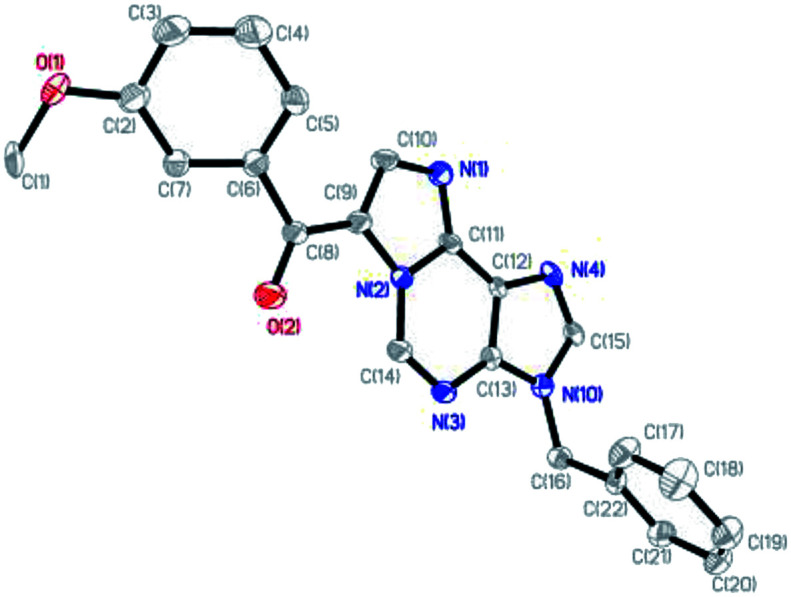
ORTEP diagram for compound 2n. Thermal ellipsoids are drawn at the 30% probability level.

To demonstrate the generality of this transformation, various substituted *N*^6^-propargyl-adenines were subjected to the optimized conditions. Firstly, the effect of aromatic ring bearing different R substituents attached to alkyne was evaluated. A series of halogens including F, Cl and Br were compatible with this cascade reaction and the desired products were generated in good yields (2b–2d). It was found that substrates bearing electron-withdrawing substituents (CN, NO_2_, CF_3_*etc.*) afforded product in higher yield than the substrate bearing electron-donating substitutes (Me, OMe, phenyl *etc.*). However, the strong electron-donating group substituted at the *para* position of the alkyne remarkably retarded the reaction (2m, trace). The electron-donating group at the *meta* position also significantly reduced the reaction yield (2n, 56%). Steric hindered substrates proved to be not suitable for the reaction and only low yields were obtained (2k, 2o and 2q). Furthermore, the effect of the substituent attached on purine, including *N*^7^-benzyl and *N*^9^-Ts substituted substrates also gave the corresponding 1,*N*^6^-ethenoadenines in good yields (2r, 65%; 2s, 83%). Fortunately, a single crystal suitable for X-ray crystallography of 2n was obtained and its structure was unambiguously confirmed in Fig. 2, which further verified the proposed structure.

Next, substrate scope was extended to ribose nucleoside substrates 1t–1w under the optimized reaction condition (Table 3). The corresponding 1,*N*^6^-ethenoadenosines were also obtained in good yields (2t–2w). Their UV and fluorescent spectroscopies were recorded. From UV spectrum, they showed a unique adsorption at 340 nm (see ESI†). Furthermore, fluorescence spectrum of 2w showed emission wave at 437 nm and excitation at 270 nm, which is very useful for nucleic acid research when incorporated into oligonucleotides. The related work is on-going and will be reported in due course.

**Table 3:** Metal-free cascade cyclization–oxidation of *N*^6^-propargyl-adenosines^a^

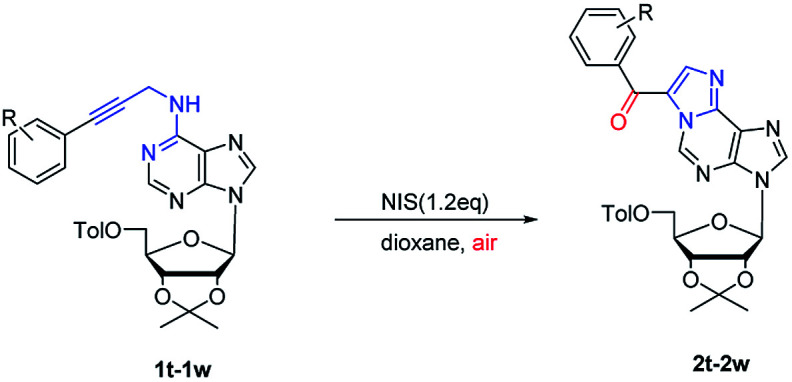
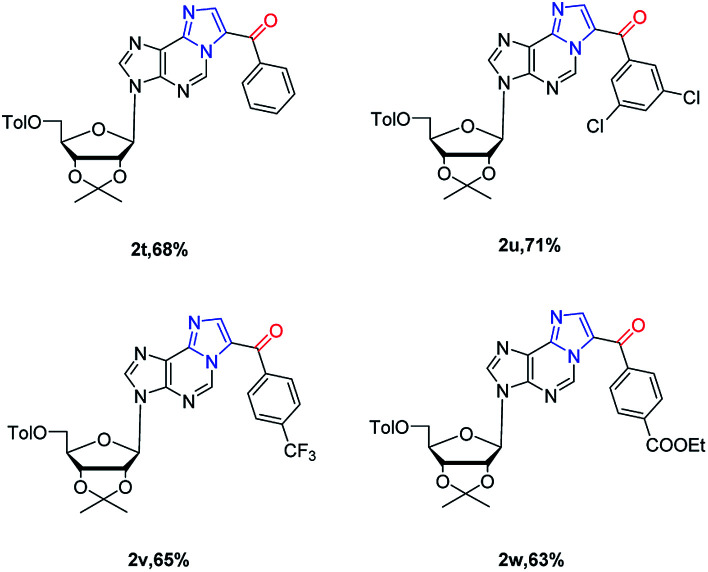

a Reaction conditions: 0.1 mmol 1t–1w, 1.2 eq. NIS, in 2.0 mL dioxane under air; isolated yields.

To gain insights into the reaction mechanism, we revisited the optimization process. We observed three clear facts: (1) the oxygen is essential for this transformation. (2) Other NXS except for NIS cannot afford the desired products in good yield. (3) Retarded reaction was observed under dark environment. Considering NIS could generate iodine radical, we assumed that the reaction might be a radical mechanism, which is contrary to our initially proposed mechanism through iodonium intermediate.

In order to test our hypothesis, control experiments were performed as shown in Scheme 2. When the radical inhibitor butylated hydroxytoluene (BHT) was added to the reaction, we observed that the reaction was almost inhibited, which indicated that a radical intermediate maybe involved in this cascade reaction (Scheme 2a). In order to further verify the resource of oxygen atom in oxidation products, isotopic labelling experiment using ^18^O_2_ were conducted. The labeled product 2f-O^18^ can be confirmed by HRMS analysis (Scheme 2b). The result demonstrated that the oxygen atom of oxidation product 2a was utmost originated from O_2_. To the best of our knowledge, bifunctionalization of alkyne by radical reaction and aerobic aminooxygenation is rarely reported.^16^ In addition, the cyclization reaction cannot happen if the *NH*-6 was blocked by methyl group.

Based on these preliminary mechanistic studies and previous reports, a plausible mechanism for this cascade reaction is proposed as shown in Scheme 3. Initially, the reaction of the *N*^6^–H of starting material 1 with NIS gave intermediate A, which is easily hemolytic to form radical intermediate B. The tautomerism of radical intermediate B could deliver *N*-1 radical intermediate C. Then, the *N*-1 radical attack the triple bond through *5-exo-dig* configuration to afford cyclized radical intermediate D in higher yields. After oxidation of D using molecular O_2_, the resulted intermediate E rearranged to give ethenoadenine 2 after elimination of HOI.

## Conclusions

In summary, an efficient approach for the preparation of 1,*N*^6^-ethenoadenine from *N*^6^-propargyl-adenine was developed. This approach features: (1) easily prepared starting material; (2) NIS mediated radical and metal-free cascade reaction; (3) air used as an oxygen source and as the oxidant. We believe that this methodology provides a complementary synthetic approach to 1,*N*^6^-ethenoadenine architectures and the NIS mediated radical cascade reaction of *N*-propargylamine under air opens up new opportunities for the synthesis of other heterocycles.

## Experimental

### General procedure for the preparation of products

NIS (27.0 mg, 0.12 mmol) was added to a stirred solution of 1a–1w (0.1 mmol) in dioxane (2 mL) under open air atmosphere. The resulting mixture was stirred at room temperature and the progress of the reaction was monitored by thin-layer chromatography. After completion of the reaction, the mixture was quenched by slow addition of saturated sodium thiosulfate and extracted with EtOAc (3 × 5 mL). The combined organic layer was dried over anhydrous Na_2_SO_4_ and concentrated under reduced pressure. The crude product was purified by column chromatography on silica gel to give the corresponding product 2a–2w.

## Conflicts of interest

There are no conflicts to declare.

## Supplementary Material

RA-009-C9RA09198J-s001

RA-009-C9RA09198J-s002
