# Supplementary material for: Metal-free synthesis of 1,N6-ethenoadenines from N6-propargyl-adenines via NIS mediated radical cascade reaction
Source: RSC Adv. 2019 Nov 26;9(66):38897–901. doi: 10.1039/c9ra09198j (PMC9075968; doi:10.1039/c9ra09198j)
Supplement: RA-009-C9RA09198J-s001 [file RA-009-C9RA09198J-s001.pdf]

Electronic Supplementary Material (ESI) for RSC Advances.
This journal is © The Royal Society of Chemistry 2019

# Metal-free synthesis of 1,*N*[6]-ethenoadenine from *N*[6]-propargyl-adenine *via* NIS mediated radical cascade reaction

Ruchun Yang,[a,b] Si Deng,[b] Xiangyou Dong,[b] Xian-Rong Song,[b] Hu Cai[a,]* Jiang Bai,[b] Qiang Xiao[b,]*

[a] Institute of Chemistry, Nanchang University, Jiangxi Province, Nanchang 330031, China. E-mail: caihu@ncu.edu.cn

[b] Institute of Organic Chemistry, Jiangxi Science & Technology Normal University, Key Laboratory of Organic Chemistry, Jiangxi Province, Nanchang 330013, China. E-mail: xiaoqiang@tsinghua.org.cn

**Table of Contents**


| | | |
|---|---|---|
| 1 | **General Considerations** | S2 |
| 2 | **General Procedure for Cascade Cyclization-Oxidation Reaction** | S2 |
| 3 | **$^{18}O_2$ Experiment** | S2 |
| 4 | **Ultraviolet spectrum and Fluorescence emission spectrum of compound 2t-2w** | S3-S4 |
| 5 | **Characterization Data for Isolated Products 2a-2w** | S4-S11 |
| 6 | **Crystal Preparation and X-ray diffraction of analysis of compound 2n** | S12 |
| 7 | **$^{1}H$ NMR and $^{13}C$ NMR Spectra for Compounds 2a-2w** | S13-S33 |

## General Considerations

All reagents were purchased from commercial sources and used without further treatment, unless otherwise indicated. Other materials were prepared according to the literature [1]. Dioxane were dried over $CaH_2$, distilled under reduced pressure, and stored under an argon atmosphere over activated 4 Å molecular sieves in Teflon screwed Schlenk flasks before using them. $^{1}$H and $^{13}$C NMR spectra were recorded on Bruker 400 MHz spectrometers or Bruker 500 MHz spectrometers, chemical shifts are given in parts per million (ppm) relative to standard tetramethylsilane (0.00 ppm for $^{1}$ H NMR) or residual solvent peaks for $^{13}$ C NMR. HRMS was obtained using a Q-TOF instrument equipped with ESI source. Fluorescence spectra were measured with a Hitachi F-4600 fluorescence spectrophotometer. UV-vis absorption spectra were recorded on an Agilent 8453 UV/Vis spectrophotometer equipped with an MUΛ-165 UV lamp and MVL-210 visible lamp for photoirradiation. Standard column chromatography was performed on 200-300 mesh silica gel. using flash column chromatography techniques.

## General procedure for cascade cyclization-oxidation reaction

NIS (27.0 mg, 0.12 mmol) was added to a stirred solution of **1a-1w** (0.1 mmol) in dioxane (2 mL). The resulting mixture was stirred at room temperature in the air and the progress of the reaction was monitored by thin-layer chromatography. After completion of the reaction, the reaction mixture was quenched by slow addition of saturated sodium thiosulfate and extracted with EtOAc (3×5mL). The combined organic layer was dried over anhydrous $Na_2SO_4$ and concentrated under reduced pressure. The crude product was purified by column chromatography on silica gel to give the corresponding product **2a–2w**.

## $^{18}O_2$ experiment

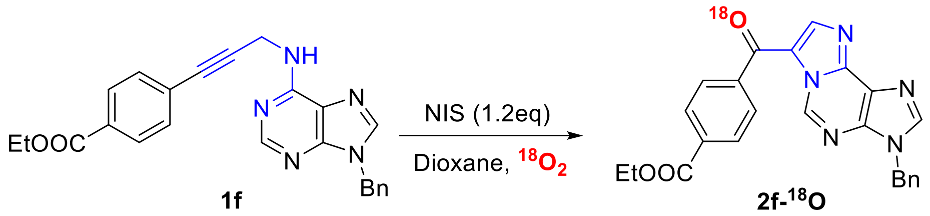


The product was detected by HRMS. HRMS (ESI, m/z): calcd for $C_{24}H_{20}N_5O_2{}^{18}O$: $[M+H]^+$ =428.1603; found: 428.1696.

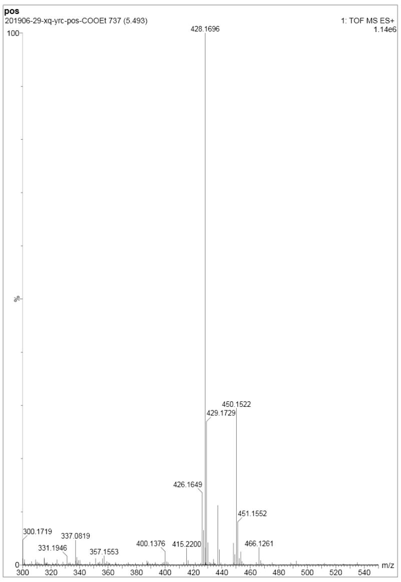


## Ultraviolet spectrum and fluorescence emission spectrum of compound 2t-2w

Ultraviolet spectrum（$1*10^{-4}$mol/L in Methanol）

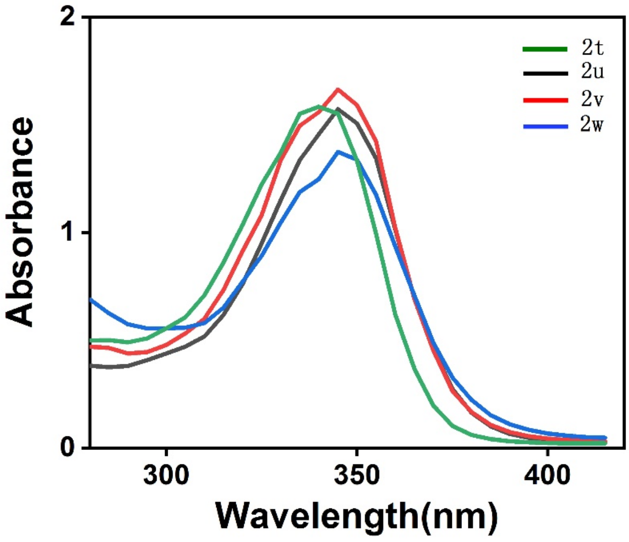


Fluorescence emission spectrum（$2*10^{-5}$mol/L in Methanol）

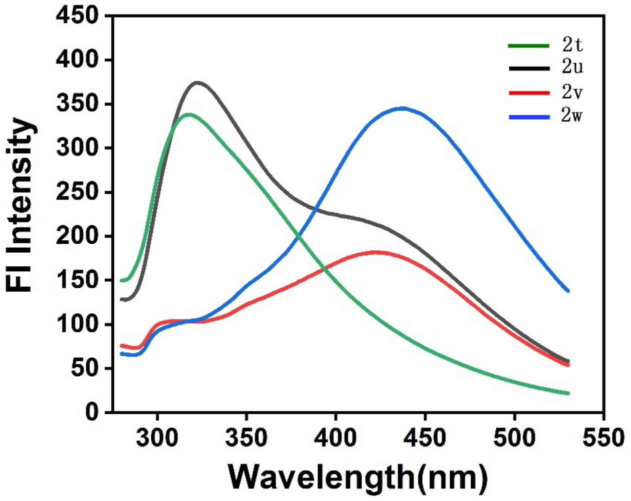


Excitation wavelength: 270nm
Emission wavelength: **2t**: 318nm, **2u**: 322nm, **2v**: 422nm, **2w**: 437nm

## Characterization Data for Isolated Products

**(3-benzyl-3H-imidazo[2,1-i] purin-7-yl) (phenyl)methanone (2a):**

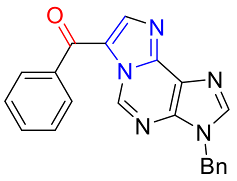


The resultant residue was purified by flash silica gel column chromatography to afford **2a** as a white solid, $^1$H NMR (500 MHz, $CDCl_3$) δ 10.36 (s, 1H), 8.24 (s, 1H), 8.09 (s, 1H), 7.91 (d, *J* = 5.4 Hz, 2H), 7.66-7.56 (m, 3H), 7.40-7.34 (m, 5H), 5.56 (s, 2H). $^{13}$C NMR (126 MHz, $CDCl_3$) δ 184.9, 146.3, 145.3, 142.1, 141.9, 138.8, 137.8, 136.1, 132.6, 129.2, 128.9, 128.8, 128.7, 127.8, 123.6, 123.2, 48.1. HRMS (ESI, m/z): calcd for $C_{21}H_{16}N_5O$: $[M+H]^+$= 354.1349; found: 354.1347.

**(3-benzyl-3H-imidazo[2,1-i] purin-7-yl) (4-chlorophenyl) methanone (2b):**

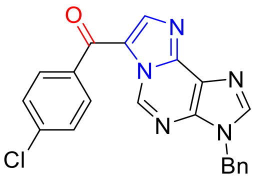


The resultant residue was purified by flash silica gel column chromatography to afford **2b** as a white solid, $^1$H NMR (400 MHz, DMSO) δ 10.14 (s, 1H), 8.70 (s, 1H), 8.34 (s, 1H), 8.00 – 7.87 (m, 2H), 7.75 – 7.62 (m, 2H), 7.42 – 7.10 (m, 6H), 5.64 (s, 2H). $^{13}$C NMR (100 MHz, DMSO) δ 182.9, 146.3, 145.1, 144.2, 142.1, 137.7*2, 137.4, 137.0, 131.3, 129.4, 129.3, 128.4, 127.9, 123.6, 123.0, 47.5. HRMS (ESI, m/z): calcd for $C_{21}H_{15}ClN_5O$: $[M+H]^+$= 388.0960; found: 388.0964

**(3-benzyl-3H-imidazo[2,1-i] purin-7-yl) (4-bromophenyl) methanone (2c):**

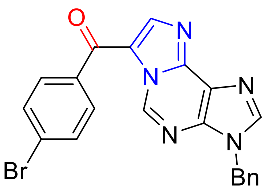

The resultant residue was purified by flash silica gel column chromatography to afford **2c** as a white solid, $^{1}$H NMR (400 MHz, DMSO) δ 10.13 (s, 1H), 8.70 (s, 1H), 8.33 (s, 1H), 7.94 – 7.76 (m, 4H), 7.63 – 7.28 (m, 5H), 5.64 (s, 2H). $^{13}$C NMR (100 MHz, DMSO) δ 183.1, 146.4, 145.4, 144.1, 142.0, 137.8, 137.7, 132.3, 131.4, 129.2, 128.4, 128.0, 126.8, 123.5, 123.0, 47.5. HRMS (ESI, m/z): calcd for $C_{21}H_{15}BrN_5O$: $[M+H]^+$ = 432.0454; found: 432.0458

**(3-benzyl-3H-imidazo[2,1-i] purin-7-yl) (4-fluorophenyl) methanone (2d):**

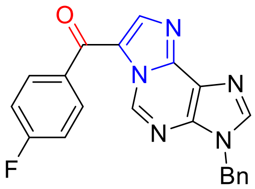

The resultant residue was purified by flash silica gel column chromatography to afford **2d** as a white solid, $^{1}$H NMR (500 MHz, $CDCl_3$) δ 10.32 (s, 1H), 8.22 (s, 1H), 8.10 (s, 1H), 7.95 (dd, *J* = 8.4, 5.4 Hz, 2H), 7.42 – 7.33 (m, 5H), 7.29 – 7.22 (m, 2H), 5.56 (s, 2H). $^{13}$C NMR (126 MHz, $CDCl_3$) δ 183.3, 165.4 (d, *J* = 254.0 Hz), 146.1, 145.5, 142.2, 141.9, 137.7, 136.0, 135.0, 134.9, 131.4 (d, *J* = 9.1 Hz), 129.8, 128.7, 127.9, 123.4 (d, *J* = 16.0 Hz), 116.0 (d, *J* = 22.0 Hz), 48.2.

$^{19}$F NMR (471 MHz, $CDCl_3$) δ -105.84.

HRMS (ESI, m/z): calcd for $C_{21}H_{15}FN_5O$: $[M+H]^+$= 32.1255; found: 372.1257

**4-(3-benzyl-3H-imidazo[2,1-i] purine-7-carbonyl) benzonitrile (2e):**

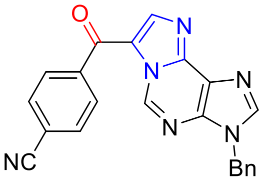

The resultant residue was purified by flash silica gel column chromatography to afford **2e** as a white solid, $^{1}$H NMR (400 MHz, $CDCl_3$) δ 10.34 (s, 1H), 8.21 (s, 1H), 8.12 (s, 1H), 8.00 (d, *J* = 7.9 Hz, 2H), 7.88 (d, *J* = 8.0 Hz, 2H), 7.34-7.38 (m, 5H), 5.57 (s, 2H). $^{13}$C NMR (126 MHz, DMSO) δ 182.6, 146.8, 145.9, 142.5, 142.3*2, 137.7, 134.9, 132.7, 129.3, 129.2, 128.8, 127.9, 123.3, 123.2, 117.9, 115.8, 48.2. HRMS (ESI, m/z): calcd for C $C_{22}H_{15}N_6O$: $[M+H]^+$= 379.1302; found: 379.1308

**ethyl 4-(3-benzyl-3H-imidazo[2,1-i] purine-7-carbonyl) benzoate (2f):**

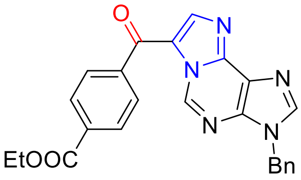

The resultant residue was purified by flash silica gel column chromatography to afford **2f** as a white solid, $^{1}H$ NMR (500 MHz, $CDCl_3$) δ 10.36 (s, 1H), 8.22-8.24 (m, 3H), 8.12 (s, 1H), 7.95 (d, *J* = 8.1 Hz, 1H), 7.42 – 7.31 (m, 5H), 5.57 (s, 1H), 4.45 (q, *J* = 7.1 Hz, 2H), 1.45 (t, *J* = 7.1 Hz, 3H). $^{13}C$ NMR (126 MHz, $CDCl_3$) δ 183.9, 165.7, 146.7, 145.6, 142.3, 142.2, 142.1, 137.6, 135.0, 133.8, 129.9, 129.2, 128.8, 127.9, 123.5, 123.3, 61.5, 48.2, 14.3. HRMS (ESI, m/z): calcd for C24H19$N_5O_3$: $[M+H]^+$ = 426.1561; found: 426.1562

**(3-benzyl-3H-imidazo[2,1-i] purin-7-yl) (4-(trifluoromethyl) phenyl) methanone (2g):**

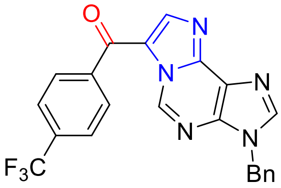


The resultant residue was purified by flash silica gel column chromatography to afford **2g** as a white solid, $^{1}H$ NMR (500 MHz, $CDCl_3$) δ 10.35 (s, 1H), 8.22 (s, 1H), 8.12 (s, 1H), 8.01 (d, *J* = 7.9 Hz, 1H), 7.84 (d, *J* = 8.0 Hz, 1H), 7.35-7.38 (m, 5H). $^{13}C$ NMR (126 MHz, $CDCl_3$) δ 183.4, 146.8, 145.7, 142.4, 142.2, 141.8, 137.7, 134.9, 133.9 (q, *J* = 32.8 Hz), 129.3, 129.2, 128.8, 127.9, 125.8 (q, *J* = 2.52 Hz), 123.6 (q, *J* = 273.4 Hz), 123.4, 123.3, 48.2. $^{19}F$ NMR (471 MHz, $CDCl_3$) δ -62.95. HRMS (ESI, m/z): calcd for $C_{22}H_{15}F_3N_5O$: $[M+H]^+$= 422.1223; found: 422.1227

**4-(3-benzyl-3H-imidazo[2,1-i] purine-7-carbonyl) benzaldehyde (2h):**

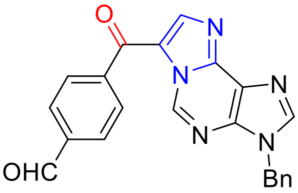


The resultant residue was purified by flash silica gel column chromatography to afford **2h** as a white solid, $^{1}H$ NMR (500 MHz, $CDCl_3$) δ 10.37 (s, 1H), 10.17 (s, 1H), 8.23 (s, 1H), 8.12 (s, 1H), 8.07 (dd, *J* = 18.1, 7.9 Hz, 4H), 7.41 – 7.33 (m, 5H), 5.57 (s, 2H). $^{13}C$ NMR (126 MHz, $CDCl_3$) δ 191.5, 183.7, 146.9, 145.7, 143.6, 142.4, 142.2, 138.6, 137.7, 134.9, 130.0, 129.4, 129.2, 128.8, 127.9, 123.4, 123.3, 48.2. HRMS (ESI, m/z): calcd for $C_{22}H_{16}N_5O_2$: $[M+H]^+$= 382.1299; found: 382.1294

**(3-benzyl-3H-imidazo[2,1-i] purin-7-yl) (4-nitrophenyl) methanone (2i):**

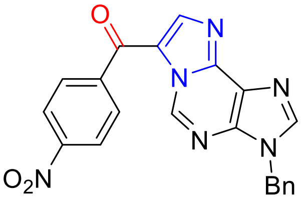


The resultant residue was purified by flash silica gel column chromatography to afford **2i** as a white solid, $^{1}H$ NMR (500 MHz, $CDCl_3$) δ 10.35 (s, 1H), 8.43 (d, *J* = 8.2 Hz, 2H), 8.22 (s, 1H), 8.13 (s, 1H), 8.06 (d, *J* = 8.3 Hz, 2H), 7.36-7.39 (m, 5H), 5.58 (s, 2H). $^{13}C$ NMR (126 MHz, $CDCl_3$) δ 182.3, 150.0, 147.1,

146.0, 143.9, 142.7, 142.3, 137.6, 134.9, 129.8, 129.3, 128.8, 127.9, 124.0, 123.4, 123.2, 47.9. HRMS (ESI, m/z): calcd for $C_{21}H_{15}N_6O_3$: $[M+H]^+$= 399.1200; found: 399.1197

**3-benzyl-3H-imidazo[2,1-i] purin-7-yl) (3,5-dichlorophenyl) methanone (2j):**

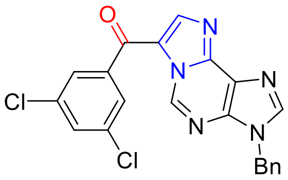


The resultant residue was purified by flash silica gel column chromatography to afford **2j** as a white solid, $^1H$ NMR (400 MHz, $CDCl_3$) δ 10.29 (s, 1H), 8.25 (s, 1H), 8.11 (s, 1H), 7.75 (s, 2H), 7.61 (s, 1H)， 7.40 – 7.34 (m, 4H), 5.56 (s, 2H). $^{13}C$ NMR (100 MHz, $CDCl_3$) δ 181.5, 146.8, 145.9, 142.5, 142.2, 141.2, 137.7, 136.7, 134.9, 132.2, 129.2, 128.8, 127.9, 127.2, 123.3, 123.0, 48.0. HRMS (ESI, m/z): calcd for $C_{21}H_{14}Cl_2N_5O$: $[M+H]^+$= 422.0570; found: 422.0566

**(3-benzyl-3H-imidazo[2,1-i] purin-7-yl) (2-chlorophenyl) methanone (2k):**

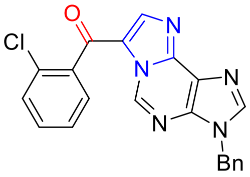


The resultant residue was purified by flash silica gel column chromatography to afford **2k** as a white solid, $^1H$ NMR (500 MHz, $CDCl_3$) δ 10.40 (s, 1H), 8.11 (s, 1H), 7.94 (s, 1H), 7.57 – 7.46 (m, 3H), 7.45 – 7.32 (m, 6H), 5.57 (s, 2H). $^{13}C$ NMR (126 MHz, $CDCl_3$) δ 183.1, 147.7, 145.8, 142.4, 142.2, 137.9, 137.7, 135.0, 131.7, 131.6, 130.6, 129.2*2, 128.8, 127.9, 126.8, 123. 9, 123.3, 48.2. HRMS (ESI, m/z): calcd for $C_{21}H_{15}ClN_5O$: $[M+H]^+$=388.0960; found: 388.0961

**(3-benzyl-3H-imidazo[2,1-i] purin-7-yl) (p-tolyl) methanone (2l):**

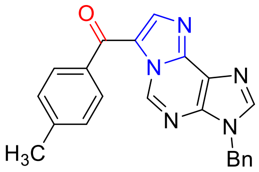


The resultant residue was purified by flash silica gel column chromatography to afford **2l** as a white solid, $^1H$ NMR (400 MHz, $CDCl_3$) δ 10.33 (s, 1H), 8.22 (s, 1H), 8.07 (s, 1H), 7.82 (d, *J* = 7.2 Hz, 2H), 7.39 – 7.31 (m, 7H), 5.55 (s, 2H), 2.47 (s, 3H). $^{13}C$ NMR (100 MHz, $CDCl_3$) δ 184.7, 145.9, 145.2, 143.3, 142.0, 141.8, 137.8, 136.1, 135.1, 129.4, 129.2, 129.1, 128.7, 127.9, 123.7, 123.3, 48.1, 21.7. HRMS (ESI, m/z): calcd for $C_{22}H_{18}N_5O$: $[M+H]^+$= 368.1506; found: 368.1503

**3-benzyl-3H-imidazo[2,1-i] purin-7-yl) (3-methoxyphenyl) methanone (2n):**

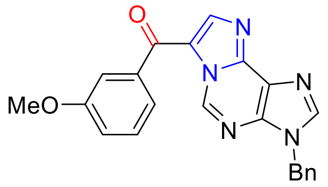

The resultant residue was purified by flash silica gel column chromatography to afford **2n** as a white solid, $^{1}$H NMR (400 MHz, $CDCl_3$) δ 10.34 (s, 1H), 8.26 (s, 1H), 8.08 (s, 1H), 7.49 – 7.32 (m, 8H), 7.21 – 7.16 (m, 1H), 5.56 (s, 2H), 3.89 (s, 3H). $^{13}$C NMR (100 MHz, CDCl3) δ 184.6, 159.8, 146.3, 145.4, 142.1, 141.9, 140.1, 137.8, 136.1, 129.7, 129.2, 128.7, 127.9, 123.6, 123.3, 121.4, 118.8, 113.6, 55.6, 48.1. HRMS (ESI, m/z): calcd for $C_{22}H_{19}N_5O_2$: $[M+H]^+$= 384.1455; found: 384.1459

**[1,1'-biphenyl]-4-yl(3-benzyl-3H-imidazo[2,1-i] purin-7-yl) methanone (2p):**

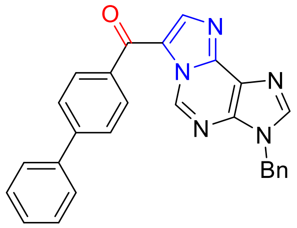

The resultant residue was purified by flash silica gel column chromatography to afford **2p** as a white solid, $^{1}$H NMR (500 MHz, $CDCl_3$) δ 10.38 (s, 1H), 8.31 (s, 1H), 8.10 (s, 1H), 8.01 (d, *J* = 7.9 Hz, 2H), 7.79 (d, *J* = 7.9 Hz, 2H), 7.69 (d, *J* = 7.6 Hz, 2H), 7.51 (t, *J* = 7.5 Hz, 2H), 7.44 – 7.33 (m, 6H), 5.57 (s, 1H). $^{13}$C NMR (126 MHz, $CDCl_3$) δ 184.4, 146.2, 145.4, 145.3, 142.1, 141.8, 139.8, 137.8, 137.4, 136.0, 129.6, 129.2, 129.0, 128.7, 128.2, 127.8, 127.4, 127.3, 123. 7, 123.3, 48.1. HRMS (ESI, m/z): calcd for $C_{27}H_{20}N_5O$: $[M+H]^+$= 430.1662; found: 430.1657

**(3-benzyl-3H-imidazo[2,1-i] purin-7-yl) (naphthalen-1-yl) methanone (2q):**

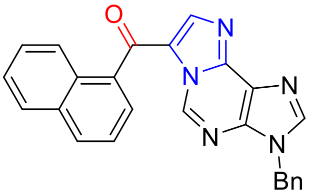

The resultant residue was purified by flash silica gel column chromatography to afford **2q** as a white solid, $^{1}$H NMR (400 MHz, $CDCl_3$) δ 10.53 (s, 1H), 8.26 – 8.21 (m, 1H), 8.10 (s, 1H), 8.06 (d, *J* = 8.3 Hz, 1H), 8.01 (s, 1H), 7.98 – 7.89 (m, 1H), 7.80 (d, *J* = 7.0 Hz, 1H), 7.57-7.60 (m, 3H), 7.40 – 7.34 (m, 5H). $^{13}$C NMR (126 MHz, $CDCl_3$) δ 186.0, 147.3, 145.7, 142.2, 142.1, 137.9, 136.2, 136.1, 133.9, 131.7, 130.7, 129.2, 128.8, 128.6, 127.9, 127.5, 127.4, 126.7, 125.2, 125.1, 124.4, 123.4, 48.2. HRMS (ESI, m/z): calcd for $C_{25}H_{18}N_5O$: $[M+H]^+$ = 404.1506; found: 404.1513

**phenyl(3-tosyl-3H-imidazo[2,1-i] purin-7-yl) methanone (2r):**

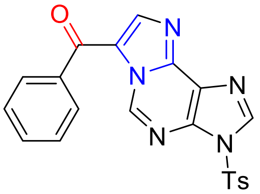


The resultant residue was purified by flash silica gel column chromatography to afford **2r** as a white solid, $^{1}H$ NMR (500 MHz, $CDCl_3$) δ 10.39 (s, 1H), 8.58 (s, 1H), 8.24 (s, 1H), 8.20 (d, *J* = 8.4 Hz, 2H), 7.89 (d, *J* = 7.7 Hz, 2H), 7.65 (t, *J* = 7.4 Hz, 1H), 7.57 (t, *J* = 7.7 Hz, 1H), 7.40 (d, *J* = 8.3 Hz, 1H), 2.44 (s, 1H). $^{13}C$ NMR (126 MHz, $CDCl_3$) δ 184.8, 147.2, 146.0, 144.3, 140.1, 139.6, 138.9, 138.3, 133.7, 132.8, 130.3, 128.9, 128.8*2, 124.7, 123.8, 21.8. HRMS (ESI, m/z): calcd for $C_{21}H_{16}N_5O_3S$: $[M+H]^+$= 418.0968; found: 418.0966

**(1-benzyl-1H-imidazo[2,1-i] purin-7-yl) (phenyl) methanone (2s):**

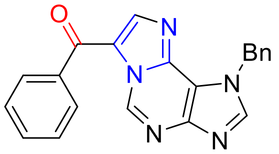


The resultant residue was purified by flash silica gel column chromatography to afford **2s** as a white solid, $^{1}H$ NMR (400 MHz, $CDCl_3$) δ 10.34 (s, 1H), 10.34 (s, 1H), 8.17 (s, 1H), 8.10 (s, 1H), 7.92 (d, *J* = 7.2 Hz, 2H), 7.65 (t, *J* = 7.4 Hz, 1H), 7.57 (dd, *J* = 8.3, 6.9 Hz, 2H), 7.51 – 7.24 (m, 5H), 5.85 (s, 2H). $^{13}C$ NMR (100 MHz, $CDCl_3$) δ 184.7, 150.6, 145.2, 143.0, 141.5, 138.7, 137.3, 135.1, 132.6, 129.2, 128.9, 128.8, 128.7, 128.1, 123.8, 114.4, 51.2. HRMS (ESI, m/z): calcd for $C_{21}H_{16}N_5O$: $[M+H]^+$= 354.1349; found: 354.1354

**((3aR,4R,6R,6aR)-6-(7-benzoyl-3H-imidazo[2,1-i] purin-3-yl)-2,2-dimethyltetrahydrofuro[3,4-d] [1,3] dioxol-4-yl) methyl 4-methylbenzoate (2t):**

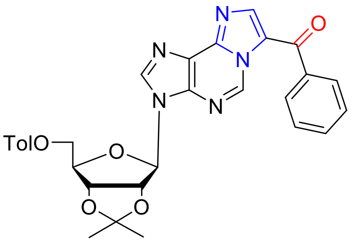


The resultant residue was purified by flash silica gel column chromatography to afford **2t** as a white solid, $^{1}H$ NMR (400 MHz, $CDCl_3$) δ 10.24 (s, 1H), 8.21 (d, *J* = 9.2 Hz, 2H), 7.91 (d, *J* = 7.7 Hz, 2H), 7.77 (d, *J* = 8.2 Hz, 2H), 7.69-7.57 (m, 3H), 7.13 (d, *J* = 8.3 Hz, 2H), 6.27 (d, *J* = 2.2 Hz, 1H), 5.58 (dd, *J* = 6.2 Hz, 2.2 Hz, 1H), 5.22 (dd, *J* = 6.3, 3.0 Hz, 1H), 4.68 – 4.65 (m, 2H), 4.51 (dd, *J* = 13.1, 5.8 Hz, 1H), 2.31 (s, 3H), 1.71 (s, 3H), 1.45 (s, 3H). $^{13}C$ NMR (100 MHz, $CDCl_3$) δ 184.7, 166.0, 146.1, 145.1, 144.1, 141.5, 140.5, 138.7, 137.8, 132.6, 129.6, 129.1, 128.9, 128.8, 126.5, 124.4, 123.6, 115.0, 91.7, 85.1, 84.5,

81.5, 63.8, 27.3, 25.5, 21.6. HRMS (ESI, m/z): calcd for $C_{30}H_{28}N_5O_6$: $[M+H]^+$= 554.2034; found: 554.2032

**((3aR,4R,6R,6aR)-6-(7-(3,5-dichlorobenzoyl)-3H-imidazo[2,1-i] purin-3-yl)-2,2-dimethyltetrahydrofuro[3,4-d] [1,3] dioxol-4-yl) methyl 4-methylbenzoate (2u):**

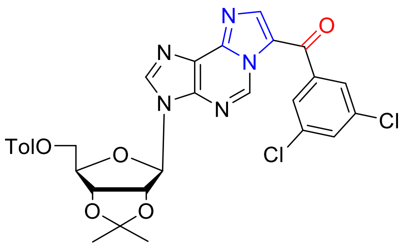


The resultant residue was purified by flash silica gel column chromatography to afford **2u** as a white solid, $^1$H NMR (400 MHz, $CDCl_3$) δ 10.18 (s, 1H), 8.23 (d, *J* = 7.8 Hz, 2H), 7.76-7.79 (m, 4H), 7.62-7.64 (m, 1H), 7.14 (d, *J* = 8.1 Hz, 2H), 6.27 (d, *J* = 2.2 Hz, 1H), 5.57 (dd, *J* = 6.2, 2.2 Hz, 1H), 5.22 (dd, *J* = 6.2, 3.0 Hz, 1H), 4.66-4.69 (m, 2H), 4.54 – 4.48 (m, 1H), 2.33 (s, 3H), 1.68 (s, 3H), 1.45 (s, 3H). $^{13}$C NMR (100 MHz, $CDCl_3$) δ 181.4, 166.0, 146.6, 145.6, 144.1, 141.8, 141.2, 141.0, 137.6, 135.8, 132.3, 129.6, 129.1, 127.2, 126.6, 124.5, 123.0, 115.1, 91.7, 85.1, 84.5, 81.5, 63.8, 27.3, 25.5, 21.7. HRMS (ESI, m/z): calcd for $C_{30}H_{26}Cl_2N_5O_6$: $[M+H]^+$=622.1255; found: 622.1255

**((3aR,4R,6R,6aR)-2,2-dimethyl-6-(7-(4-(trifluoromethyl) benzoyl)-3H-imidazo[2,1-i] purin-3-yl) tetrahydrof uro[3,4-d] [1,3] dioxol-4-yl) methyl 4-methylbenzoate (2v):**

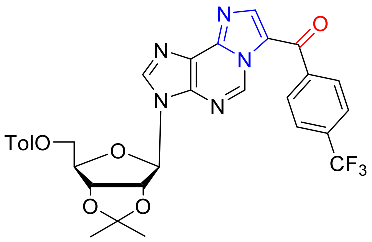


The resultant residue was purified by flash silica gel column chromatography to afford **2v** as a white solid, $^1$H NMR (400 MHz, $CDCl_3$) δ 10.23 (s, 1H), 8.21 (d, *J* = 6.9 Hz, 2H), 8.01 (d, *J* = 8.1 Hz, 2H), 7.84 (d, *J* = 8.2 Hz, 2H), 7.78 (d, *J* = 8.1 Hz, 2H), 7.14 (d, *J* = 8.0 Hz, 2H), 6.28 (d, *J* = 2.2 Hz, 1H), 5.58 (dd, *J* = 6.3, 2.2 Hz, 1H), 5.22 (dd, *J* = 6.3, 3.1 Hz, 1H), 4.669-4.66 (m, 2H), 4.54 – 4.47 (m, 1H), 2.32 (s, 3H), 1.68 (s, 3H), 1.45 (s, 3H). $^{13}$C NMR (100 MHz, $CDCl_3$) δ 183.2, 166.0, 146.6, 145.4, 144.1, 141.8*2, 140.9, 137.6, 134.1 (q, *J* = 32.8 Hz), 129.6, 129.2, 129.1, 126.6, 125.9, 124.5, 123.6 (q, *J* = 273.0 Hz), 123.4, 115.1, 91.7, 85.1, 84.5, 81.5, 63.8, 27.3, 25.5, 14.1. HRMS (ESI, m/z): calcd for $C_{31}H_{27}F_3N_5O_6$: $[M+H]^+$= 622.1908; found: 622.1908

**((3aR,4R,6R,6aR)-6-(7-(4-(ethoxycarbonyl) benzoyl)-3H-imidazo[2,1-i] purin-3-yl)-2,2-dimethyltetrahydrofuro[3,4-d] [1,3] dioxol-4-yl) methyl 4-methylbenzoate (2w):**

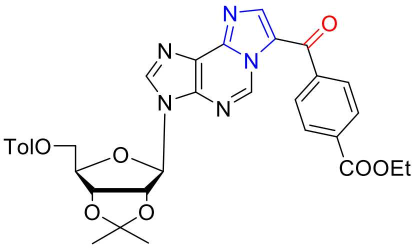


The resultant residue was purified by flash silica gel column chromatography to afford **2w** as a white solid, $^{1}H$ NMR (400 MHz, $CDCl_3$) δ 10.24 (s, 1H), 8.25 – 8.23 (m, 4H), 7.96 (d, *J* = 8.2 Hz, 2H), 7.78 (d, *J* = 8.1 Hz, 2H), 7.14 (d, *J* = 8.0 Hz, 2H), 6.28 (d, *J* = 2.1 Hz, 1H), 5.58 (dd, *J* = 6.2, 2.1 Hz, 1H), 5.23 (dd, *J* = 6.2, 3.2 Hz, 1H), 4.70 – 4.62 (m, 2H), 4.55 – 4.39 (m, 4H), 2.32 (s, 3H), 1.69 (s, 3H), 1.47-1.43 (m, 6H). $^{13}C$ NMR (125 MHz, $CDCl_3$) δ 183.8, 166.0, 165.7, 146.5, 145.2, 144.1, 142.2, 141.7, 140.7, 137.6, 133.8, 129.9, 129.5, 129.0, 128.7, 126.4, 124.4, 123.5, 115.0, 91.7, 85.0, 84.5, 81.4, 63.8, 61.5, 27.3, 25.5, 21.7, 14.3. HRMS (ESI, m/z): calcd for $C_{33}H_{32}N_5O_8$: $[M+H]^+$= 626.2245; found: 626.2247

## Notes and references


1. Li, R. L.; Liang, L.; Xie, M. S.; Qu, G. R.; Niu, H. Y.; Guo, H. M., *The Journal of organic chemistry* **2014,** *79*, 3665-70.

## Crystal preparation and X-ray diffraction analysis of compound 2n

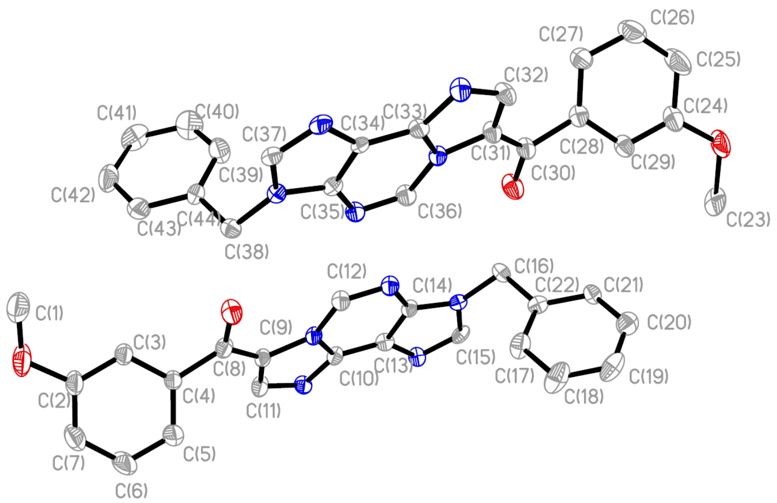


Crystal structure of compoud **2n**

# Datablock: Y

| | | | |
|---|---|---|---|
| Bond precision: | C-C = 0.0112 A | | Wavelength=0.71073 |
| Cell: | a=20.554(2) | b=7.7056(8) | c=23.283(3) |
| | alpha=90 | beta=90 | gamma=90 |
| Temperature: 296 K | | | |

| | Calculated | Reported |
|---|---|---|
| Volume | 3687.6(7) | 3687.6(7) |
| Space group | P n a 21 | P n a 21 |
| Hall group | P 2c -2n | P 2c -2n |
| Moiety formula | C22 H17 N5 O2 | ? |
| Sum formula | C22 H17 N5 O2 | C44 H34 N10 O4 |
| Mr | 383.41 | 766.81 |
| Dx,g cm-3 | 1.381 | 1.381 |
| Z | 8 | 4 |
| Mu (mm-1) | 0.093 | 0.093 |
| F000 | 1600.0 | 1600.0 |
| F000' | 1600.62 | |
| h,k,lmax | 24,9,27 | 24,9,27 |
| Nref | 6475[ 3325] | 6451 |
| Tmin,Tmax | 0.977,0.980 | |
| Tmin' | 0.977 | |

Correction method= Not given

Data completeness= 1.94/1.00 Theta(max)= 24.996

R(reflections)= 0.0671( 4559) wR2(reflections)= 0.1730( 6451)

S = 1.096 Npar= 523

## $^{1}$H and $^{13}$C NMR Data

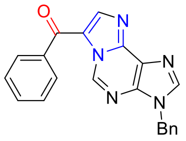


**2a**

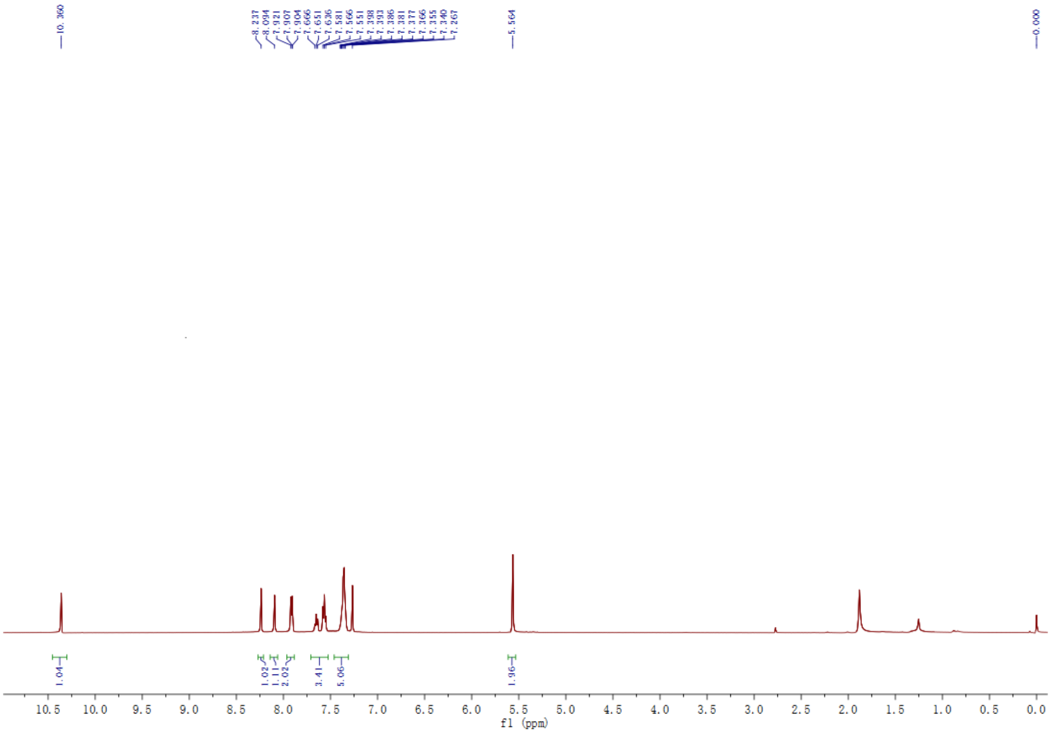

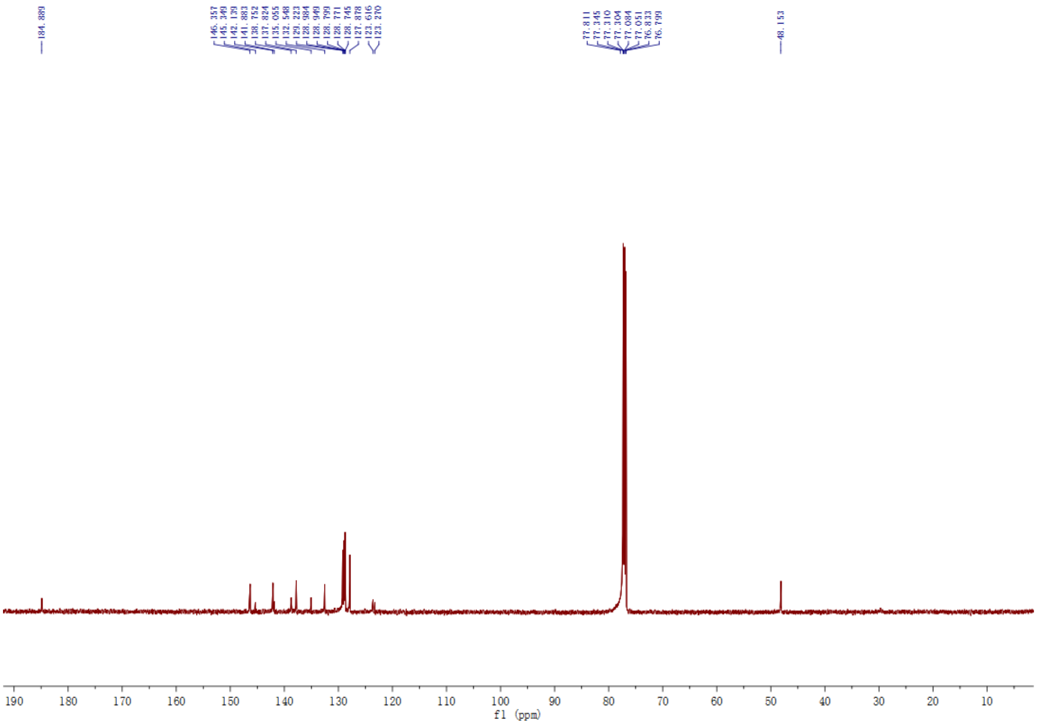

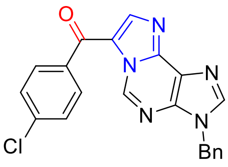

O
N
N
N
N
Cl
Bn


**2b**

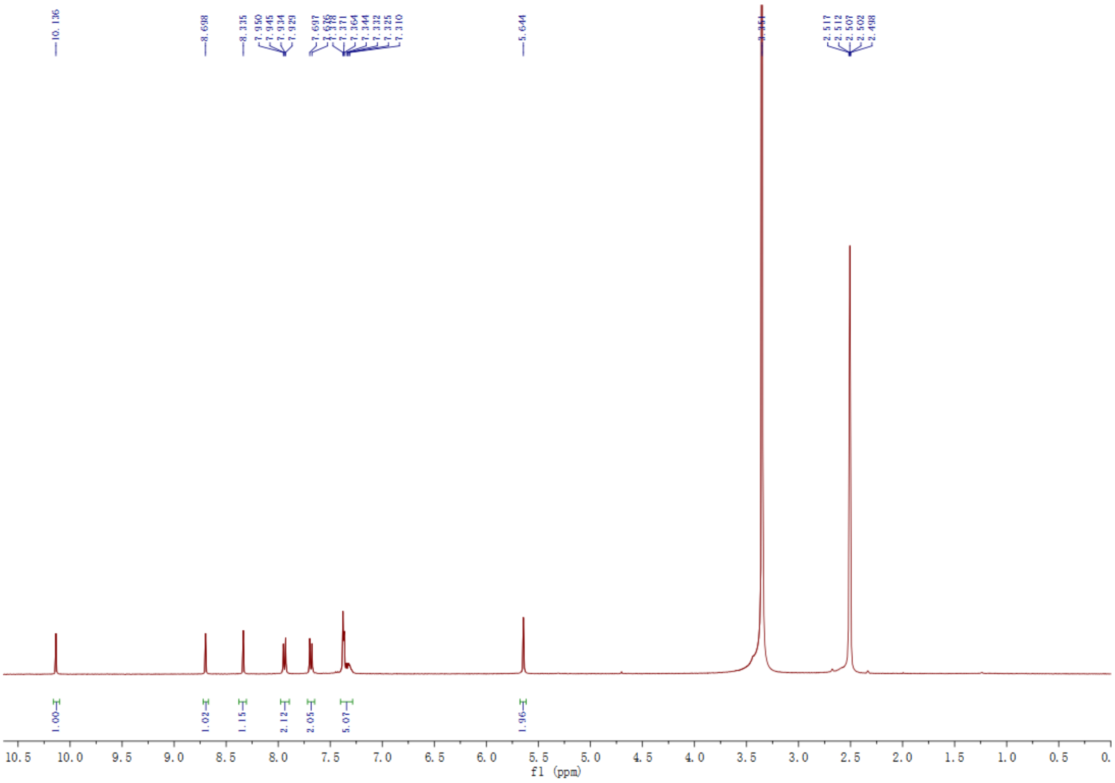

10.136
8.698
8.335
7.950
7.945
7.934
7.929
7.697
7.371
7.364
7.344
7.332
7.325
7.310
5.644
2.517
2.512
2.507
2.502
2.498
1.00
1.02
1.15
2.12
2.05
5.07
1.96
10.5 10.0 9.5 9.0 8.5 8.0 7.5 7.0 6.5 6.0 5.5 5.0 4.5 4.0 3.5 3.0 2.5 2.0 1.5 1.0 0.5 0.
f1 (ppm)


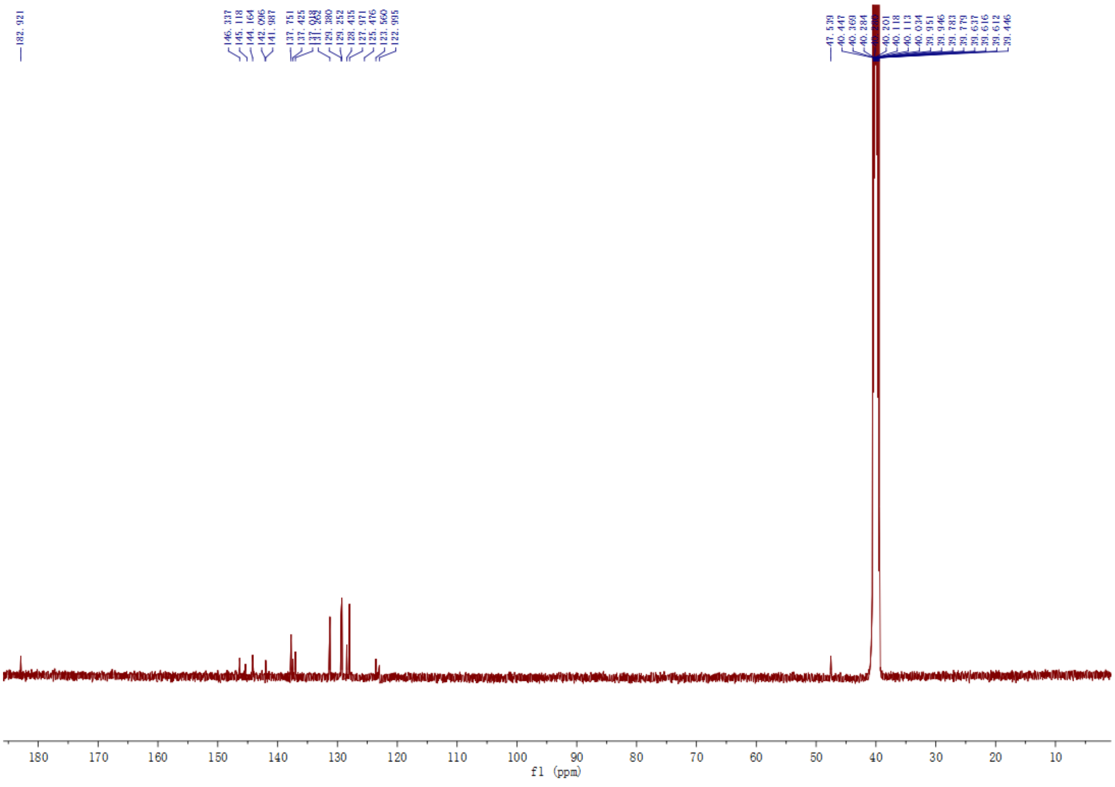

182.921
146.337
145.118
144.164
142.096
141.987
137.751
137.425
129.380
129.252
128.435
127.971
125.476
123.560
122.995
47.529
40.447
40.369
40.284
40.201
40.118
40.113
40.034
39.951
39.946
39.783
39.779
39.637
39.616
39.612
39.446
180 170 160 150 140 130 120 110 100 90 80 70 60 50 40 30 20 10
f1 (ppm)

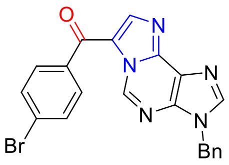

O
N
N
N
N
N
Br
Bn


**2c**

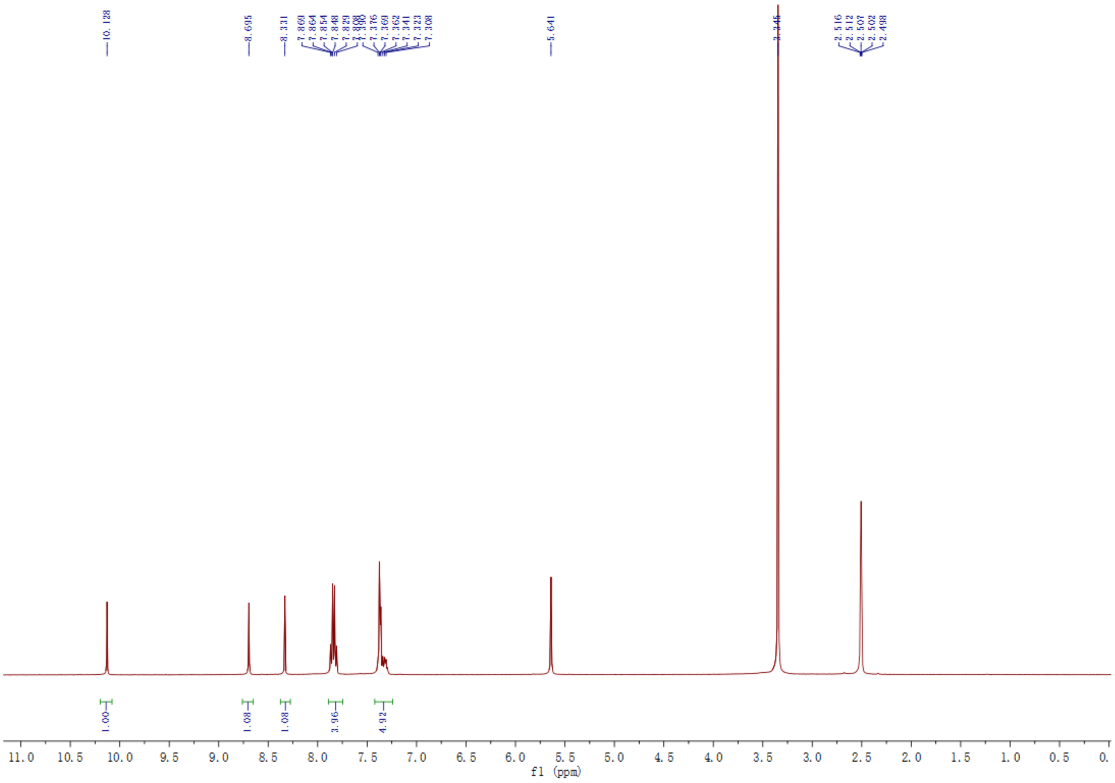

10.128
8.695
8.331
7.869
7.864
7.854
7.848
7.829
7.376
7.369
7.362
7.341
7.323
7.308
5.641
2.516
2.512
2.507
2.502
2.498
1.00
1.08
1.08
3.96
4.92
f1 (ppm)


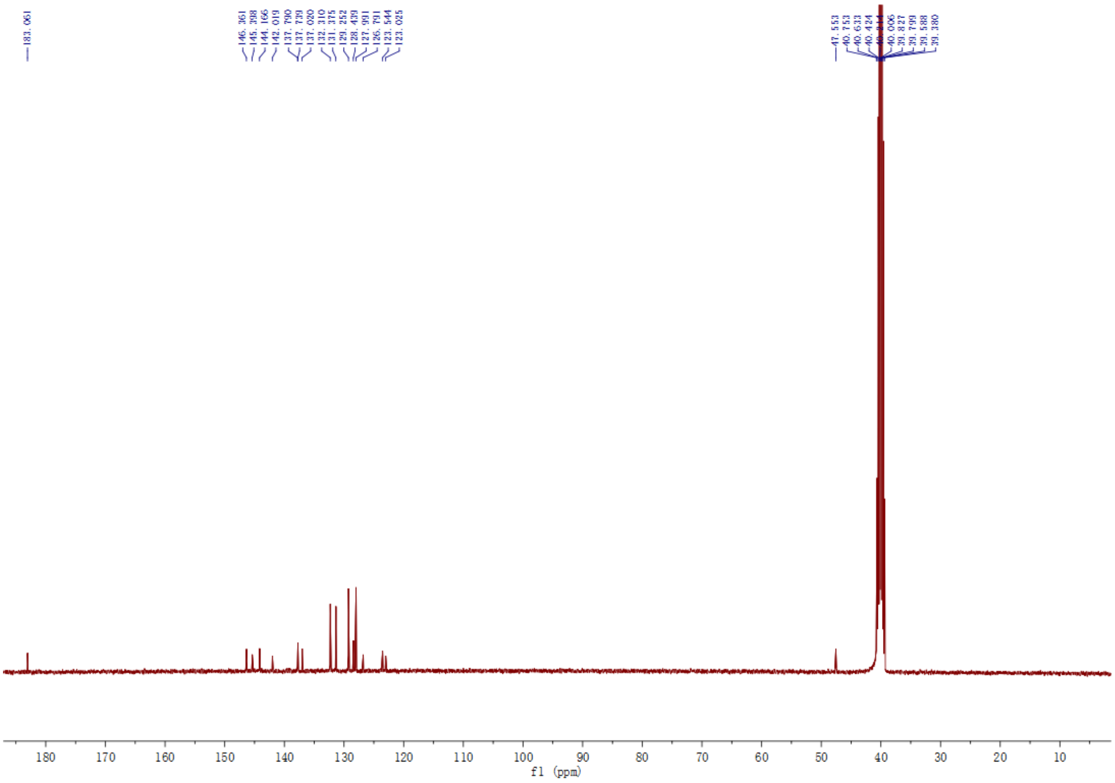

183.061
146.361
145.398
144.166
142.019
137.790
137.739
137.020
132.310
131.375
129.252
128.439
127.951
126.791
123.544
123.025
47.553
40.753
40.633
40.424
40.006
39.827
39.588
39.380
f1 (ppm)

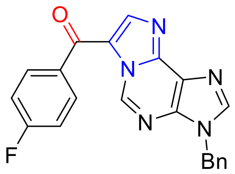


**2d**

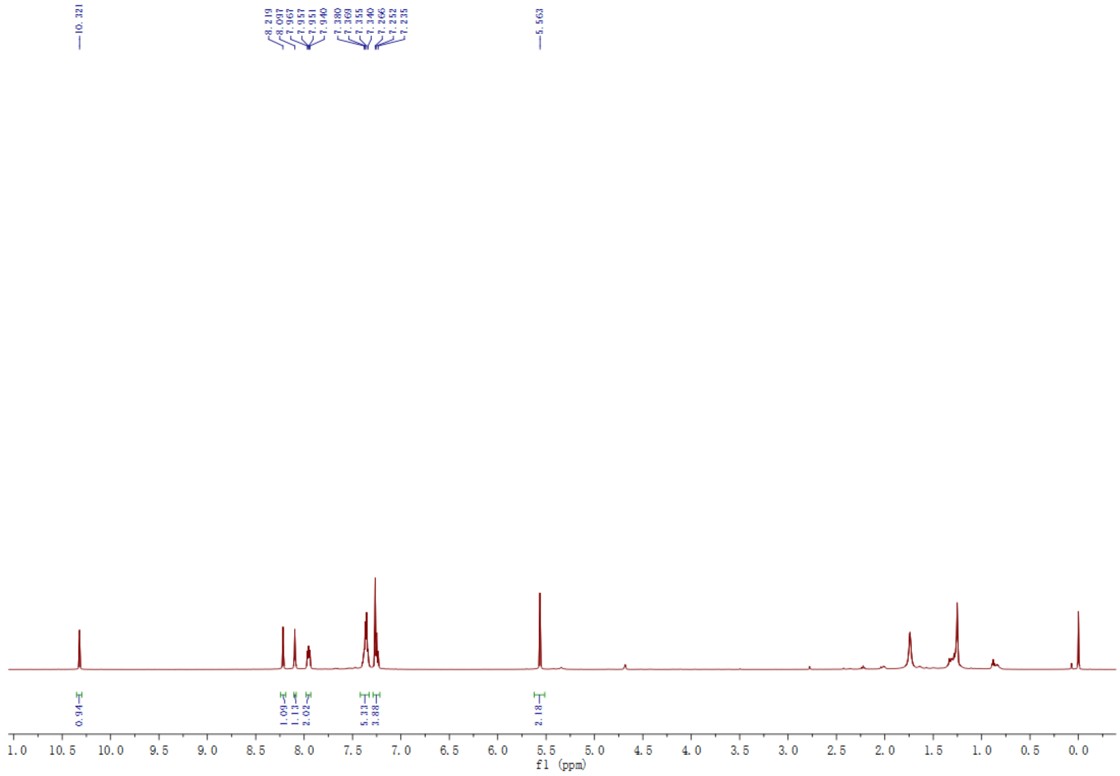

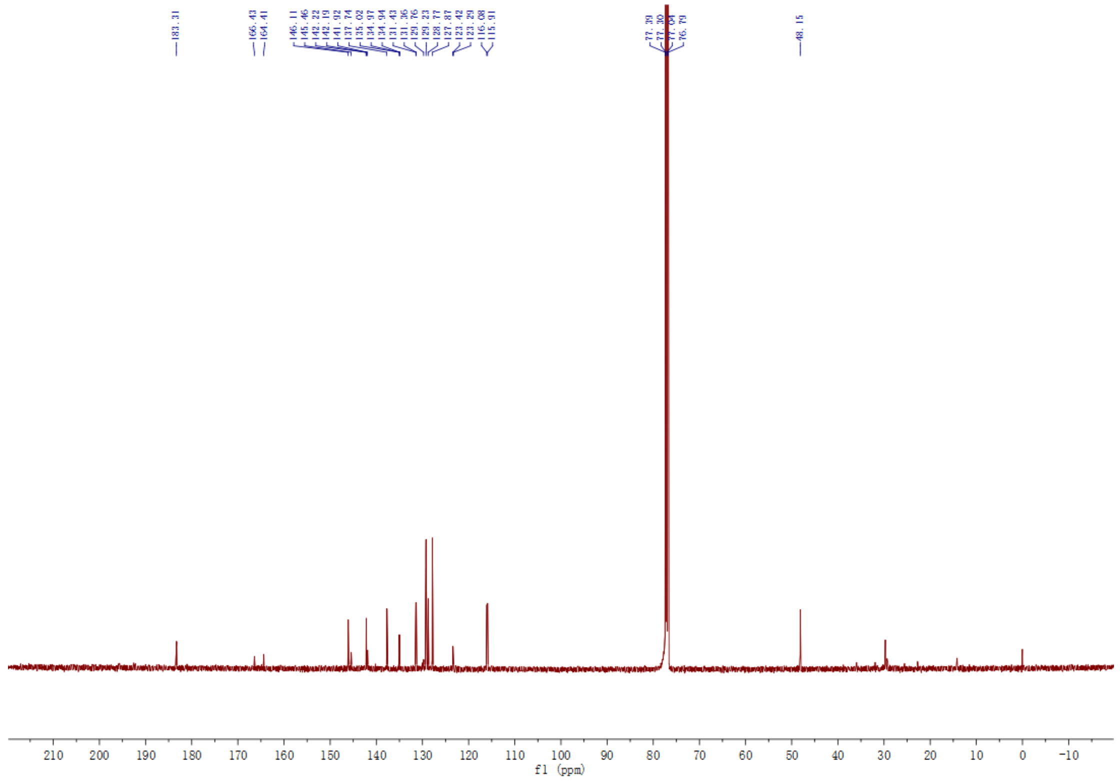

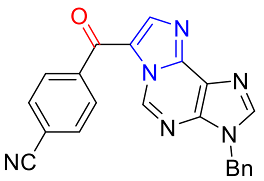

O
N
N
N
N
N
NC
Bn


**2e**

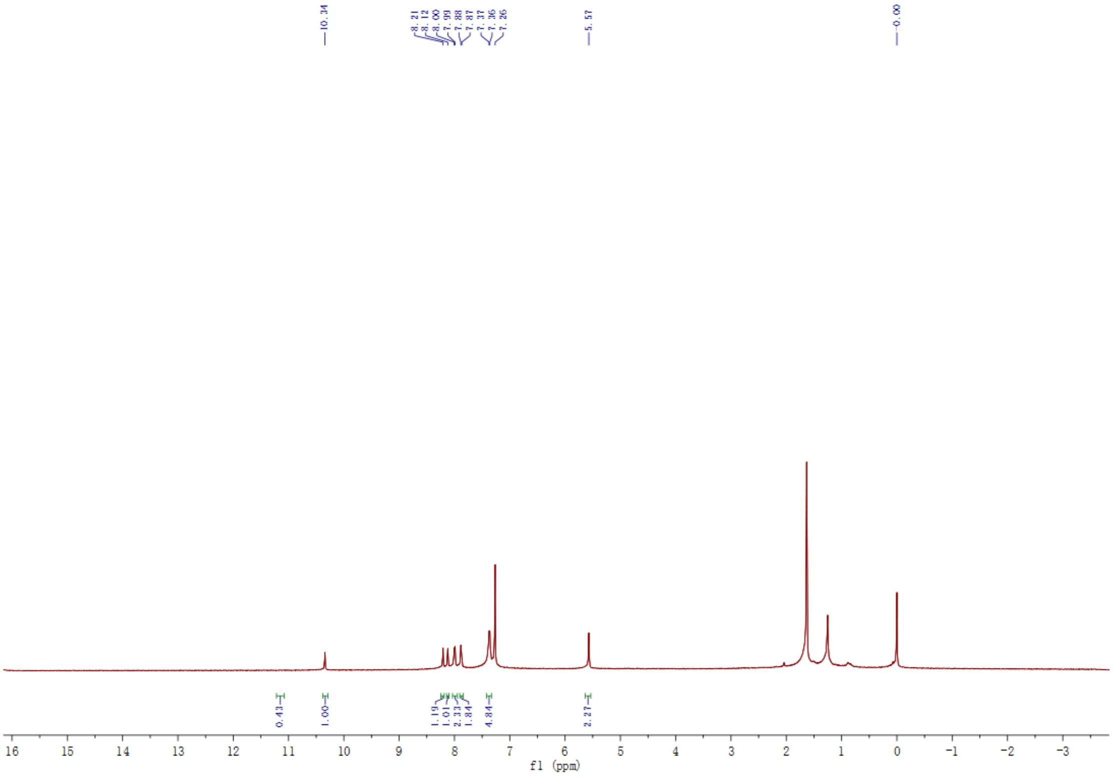

10.34
8.21
8.12
8.00
7.99
7.88
7.87
7.37
7.36
7.26
5.57
0.00
0.43
1.00
1.19
1.01
2.13
1.84
4.84
2.27
f1 (ppm)


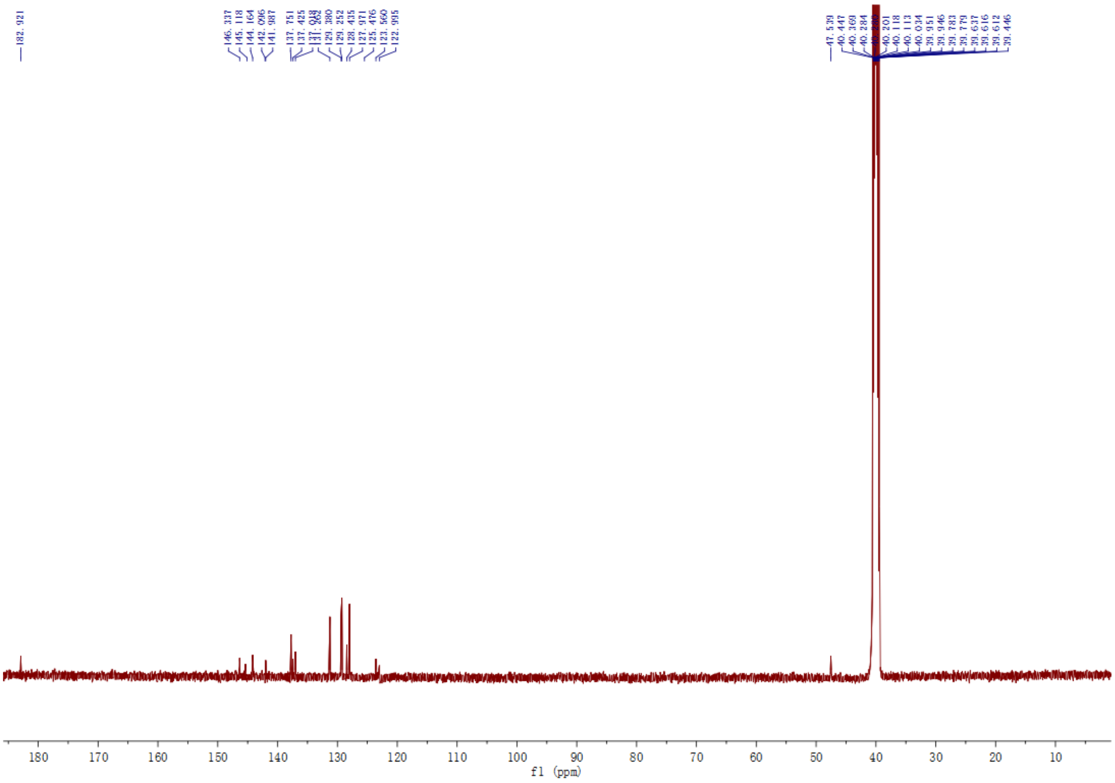

182.921
f1 (ppm)

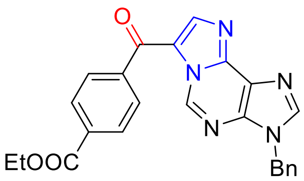
O
N
EtOOC
Bn

**2f**

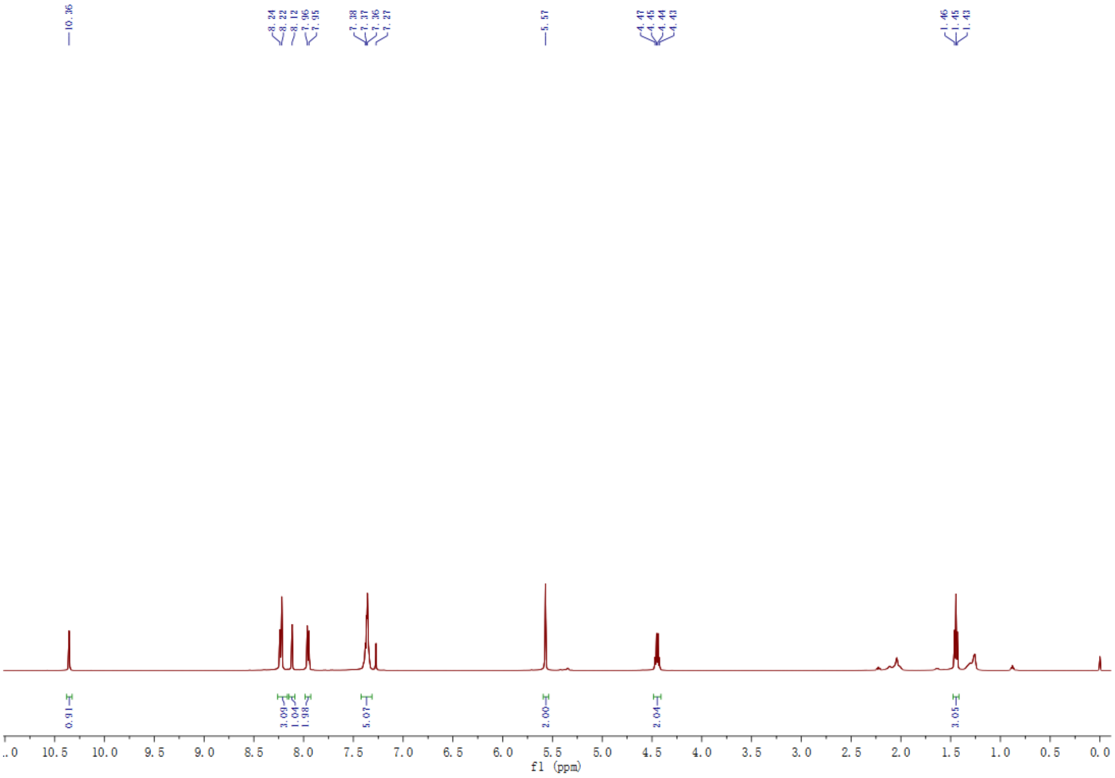
10.36
8.24
8.22
8.12
7.96
7.95
7.38
7.37
7.36
7.27
5.57
4.47
4.45
4.44
4.43
1.46
1.45
1.43
0.91
3.09
1.04
1.98
5.07
2.00
2.04
3.05
f1 (ppm)

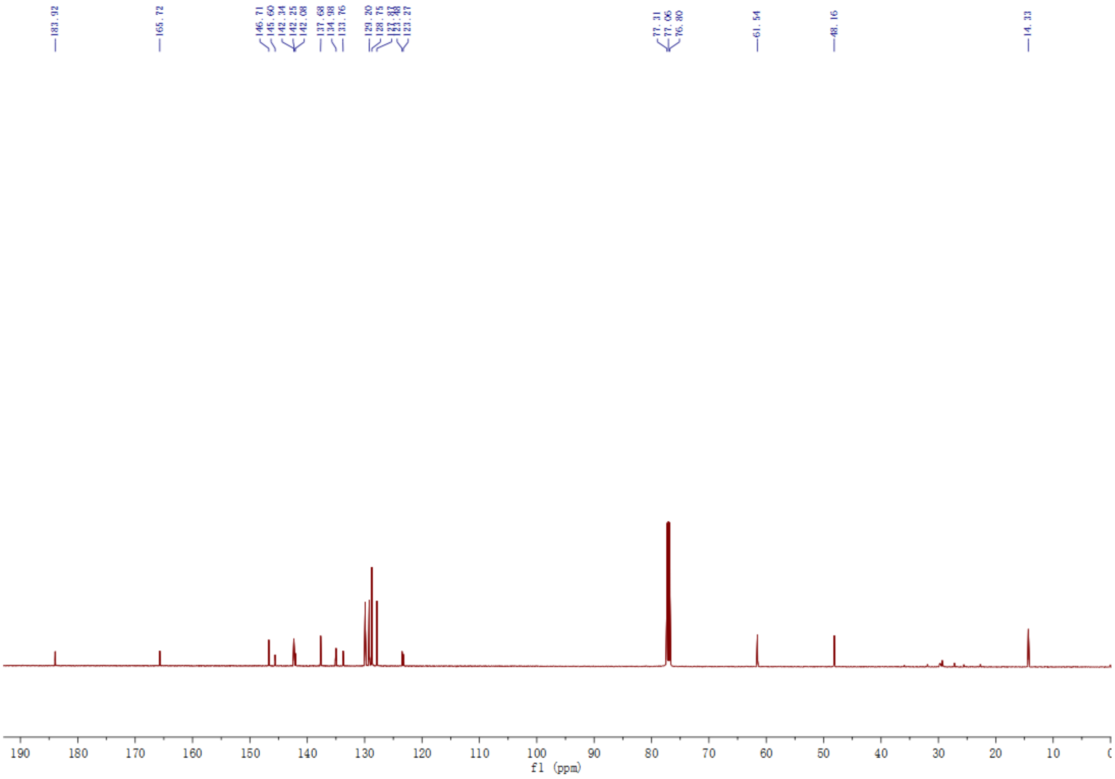
183.92
165.72
146.71
145.60
142.34
142.25
142.08
137.68
134.98
133.76
129.20
128.75
123.27
77.31
77.06
76.80
61.54
48.16
14.33
f1 (ppm)

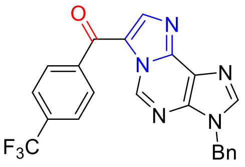
O
N
N
N
N
F3C
Bn

**2g**

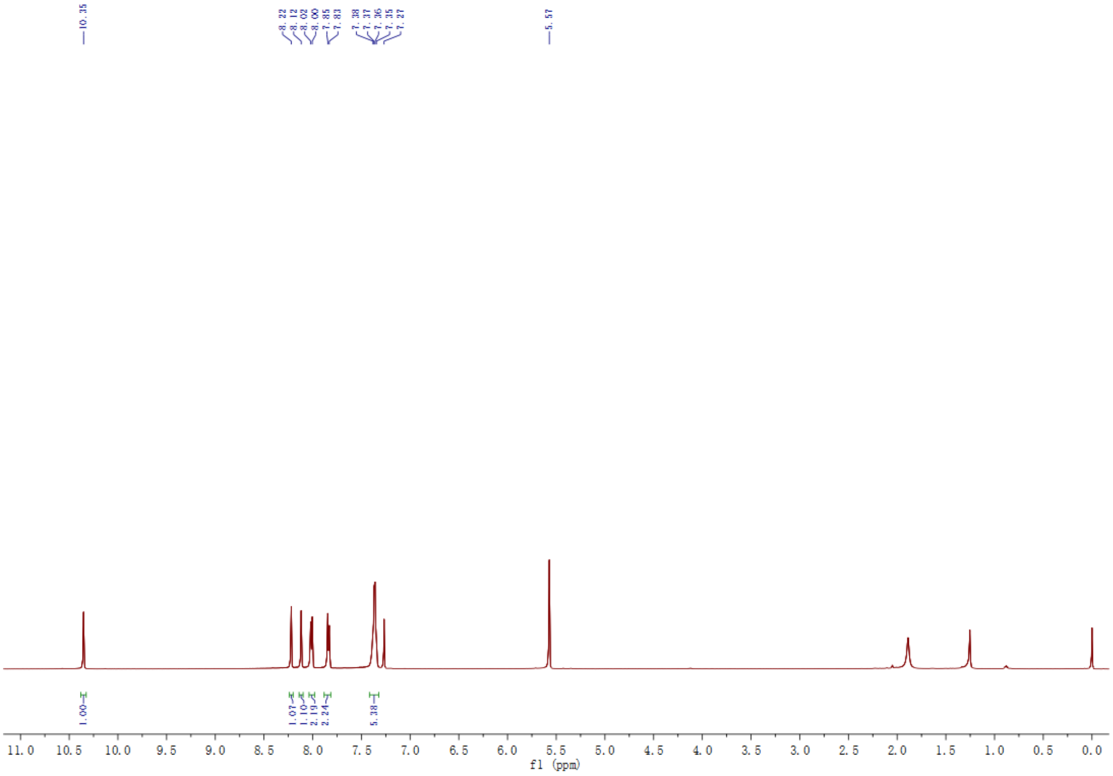
10.35
8.22
8.12
8.02
8.00
7.85
7.83
7.38
7.37
7.36
7.35
7.27
5.57
1.00
1.07
1.10
2.19
2.24
5.38
11.0
10.5
10.0
9.5
9.0
8.5
8.0
7.5
7.0
6.5
6.0
5.5
5.0
4.5
4.0
3.5
3.0
2.5
2.0
1.5
1.0
0.5
0.0
f1 (ppm)

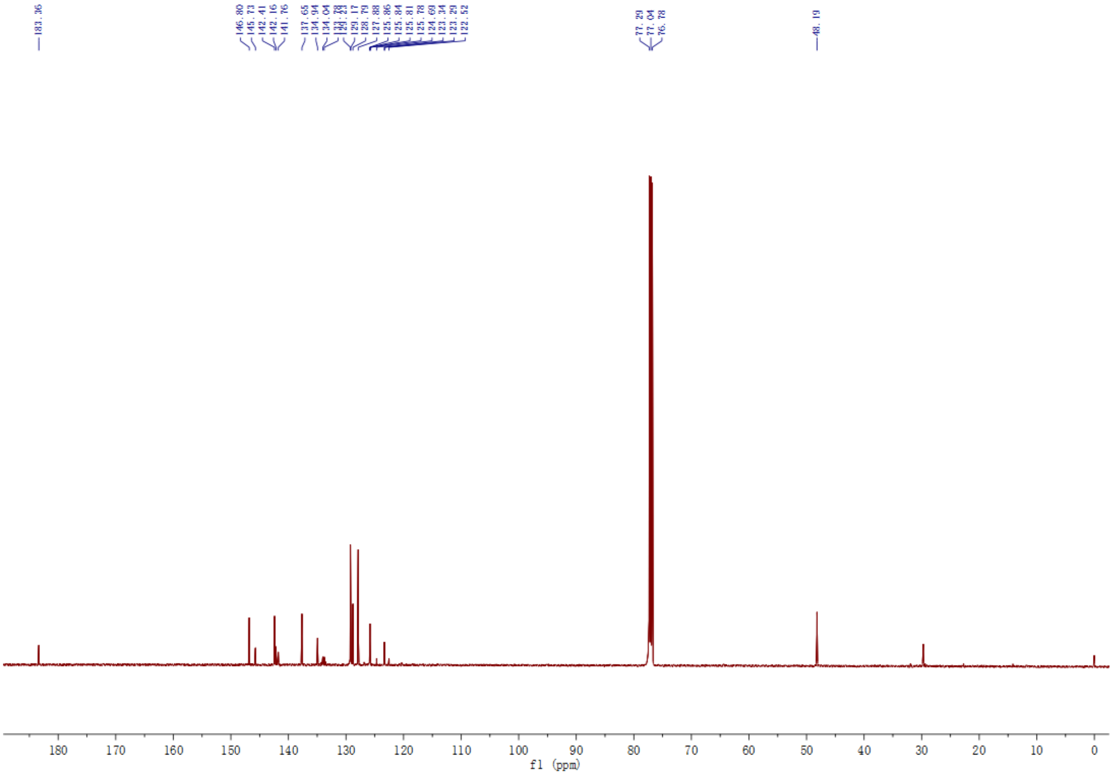
183.36
146.80
145.73
142.41
142.16
141.76
137.65
134.94
134.04
129.17
128.79
127.88
125.86
125.84
125.81
125.78
124.69
123.34
123.29
122.52
77.29
77.04
76.78
48.19
180
170
160
150
140
130
120
110
100
90
80
70
60
50
40
30
20
10
0
f1 (ppm)

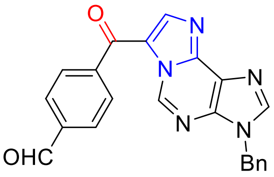


**2h**

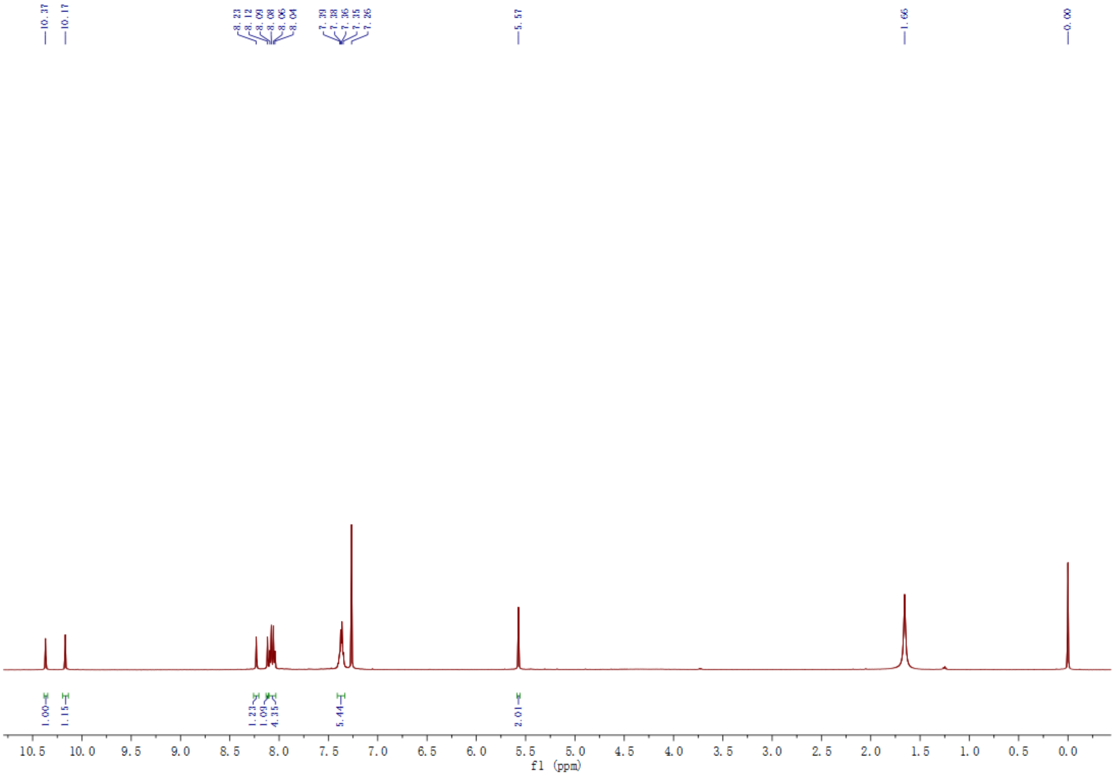

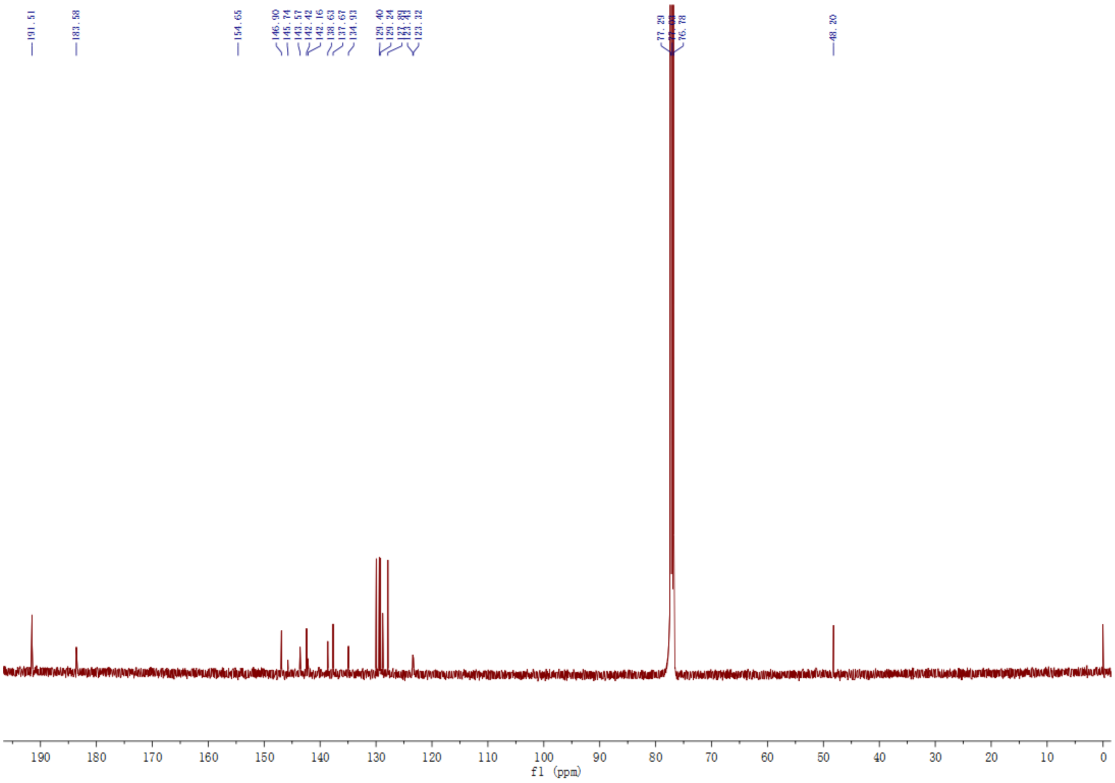

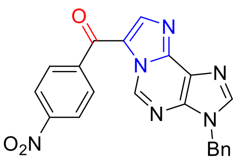
O
N
N
N
O2N
N
N
Bn

**2i**

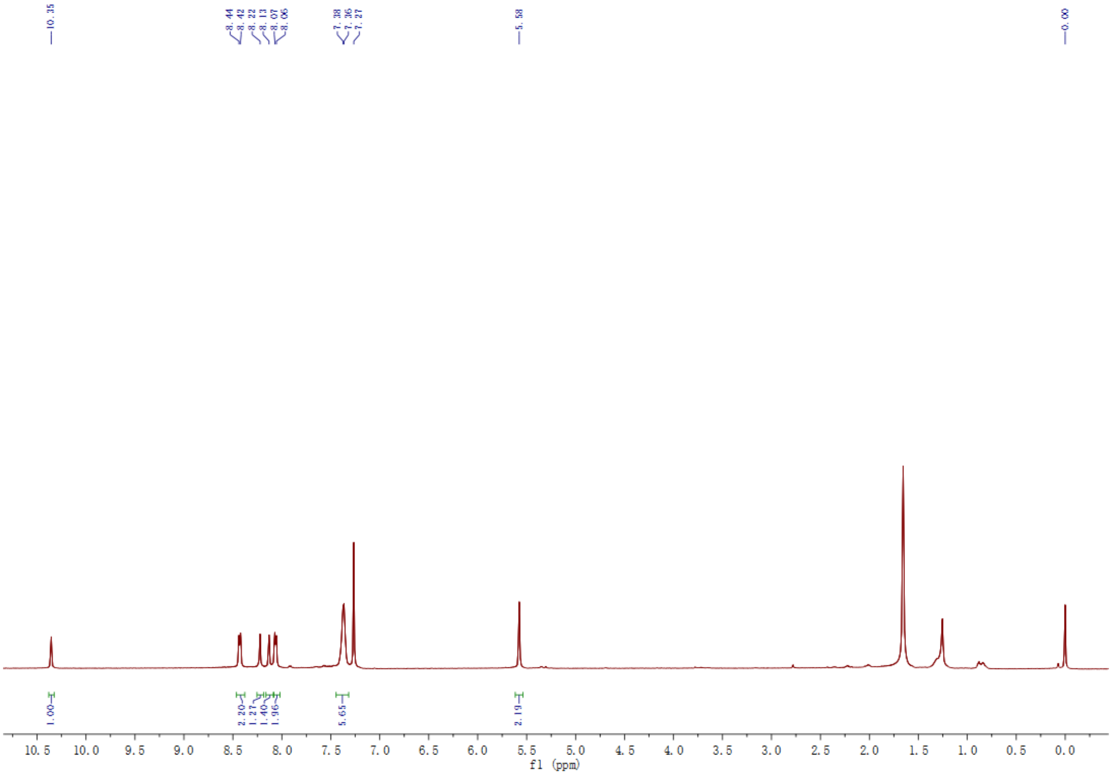
10.35
8.44
8.42
8.22
8.13
8.07
8.06
7.38
7.36
7.27
5.58
0.00
1.00
2.20
1.27
1.40
1.96
5.65
2.19
10.5 10.0 9.5 9.0 8.5 8.0 7.5 7.0 6.5 6.0 5.5 5.0 4.5 4.0 3.5 3.0 2.5 2.0 1.5 1.0 0.5 0.0
f1 (ppm)

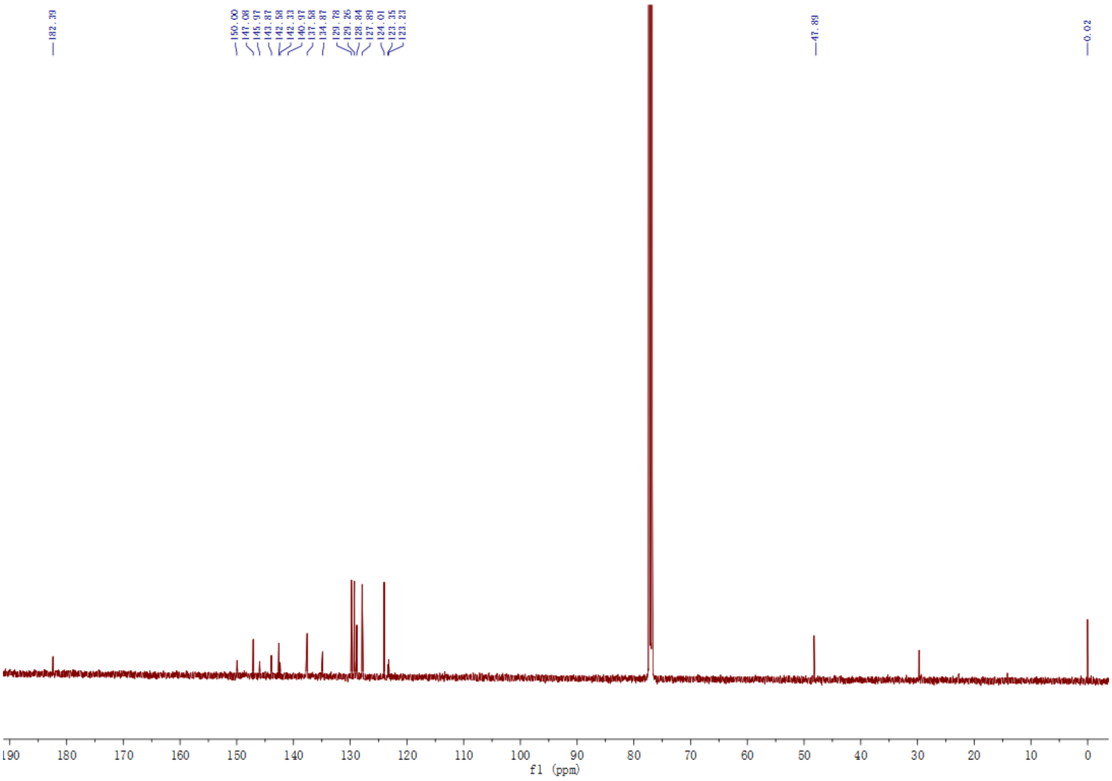
182.39
150.00
147.08
145.97
143.87
142.58
142.33
140.97
137.58
134.87
129.78
129.26
128.84
127.89
124.01
123.35
123.23
47.89
0.02
190 180 170 160 150 140 130 120 110 100 90 80 70 60 50 40 30 20 10 0
f1 (ppm)

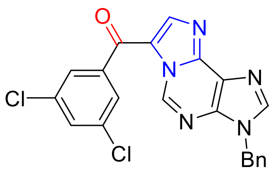
O
N
N
N
N
Cl
Cl
Bn

**2j**

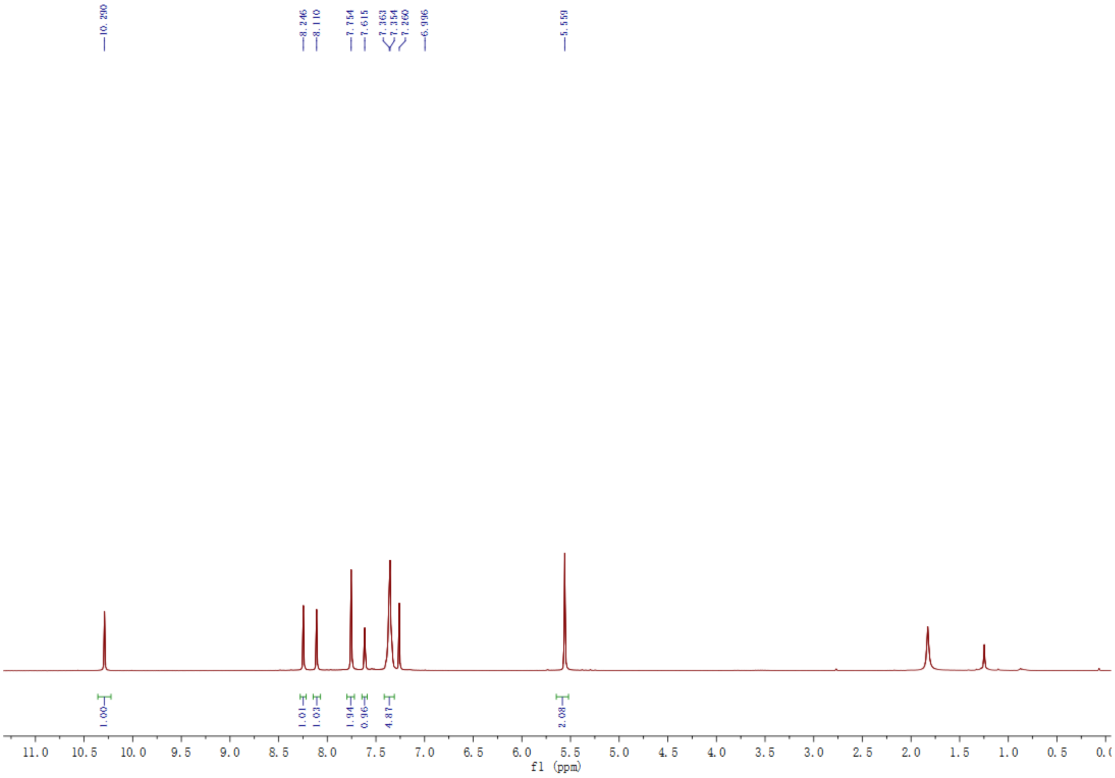
10.290
8.246
8.110
7.754
7.615
7.363
7.354
7.260
6.996
5.559
1.00
1.01
1.03
1.94
0.96
4.87
2.08
11.0 10.5 10.0 9.5 9.0 8.5 8.0 7.5 7.0 6.5 6.0 5.5 5.0 4.5 4.0 3.5 3.0 2.5 2.0 1.5 1.0 0.5
f1 (ppm)

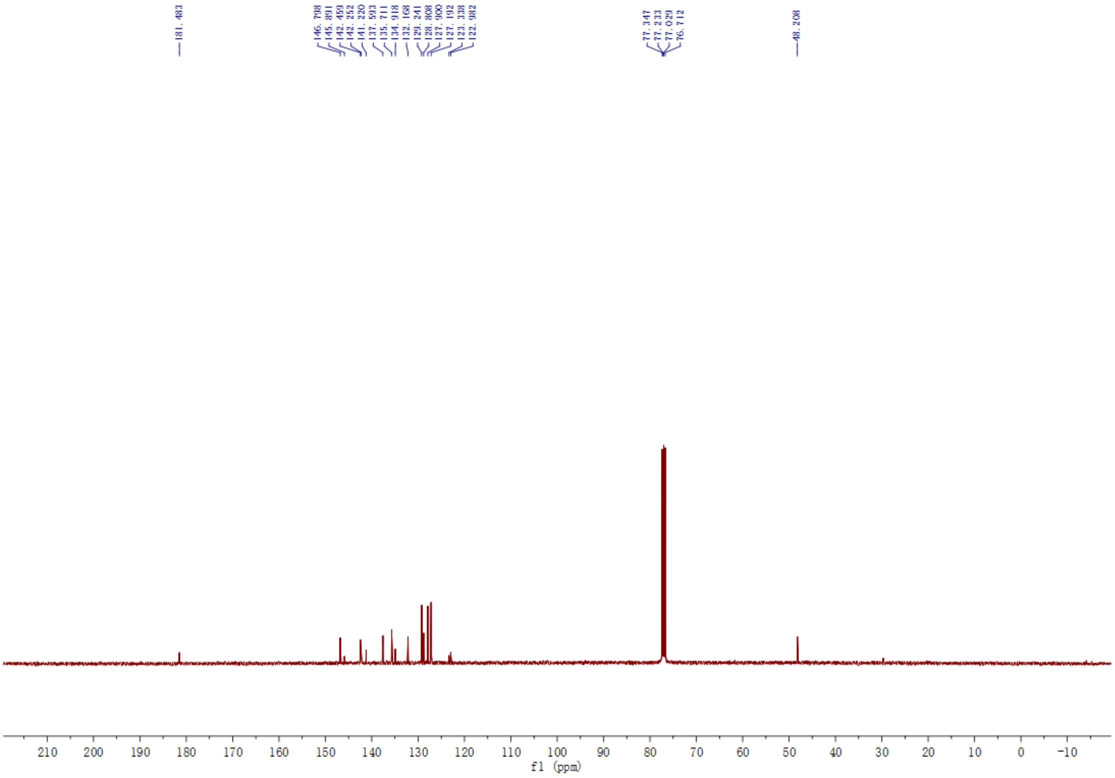
181.483
146.798
145.891
142.459
142.252
141.220
137.593
135.711
134.918
132.168
129.241
128.808
127.900
127.192
123.338
122.982
77.347
77.233
77.029
76.712
48.208
210 200 190 180 170 160 150 140 130 120 110 100 90 80 70 60 50 40 30 20 10 0 -10
f1 (ppm)

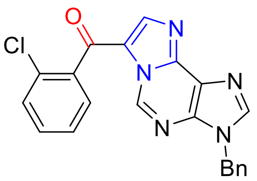

Cl
O
N
N
N
N
N
Bn


**2k**

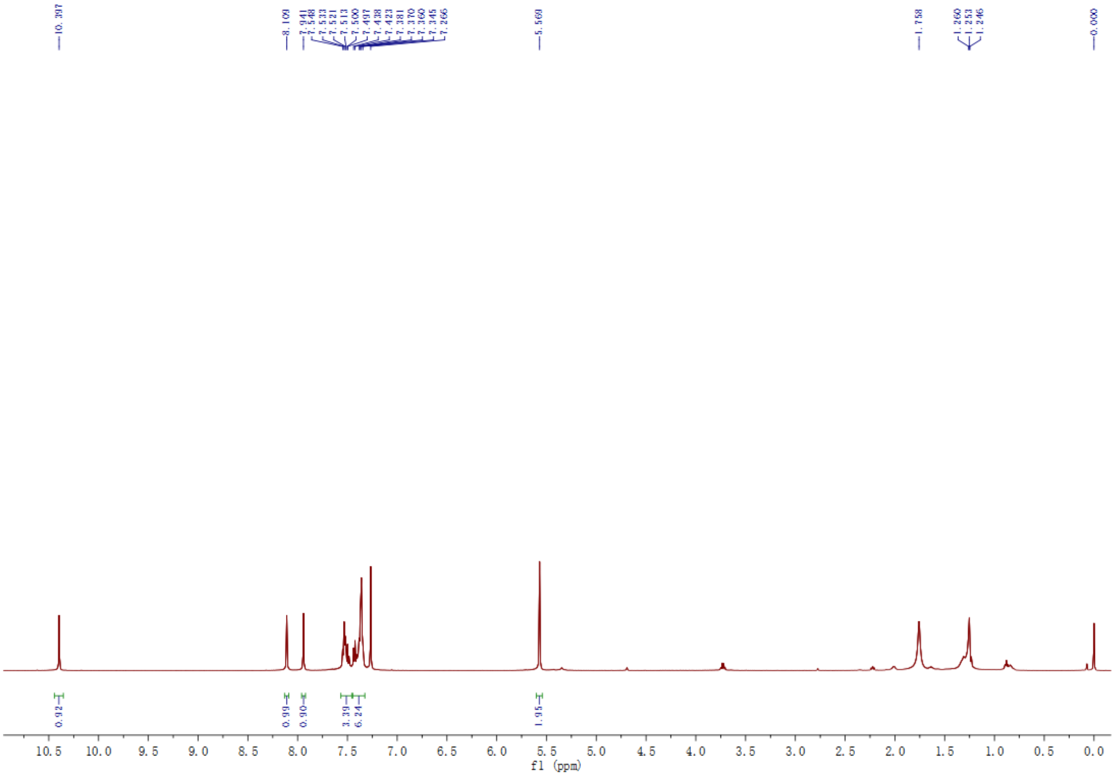

10.397
8.109
7.941
7.548
7.532
7.521
7.513
7.500
7.497
7.438
7.423
7.381
7.370
7.360
7.345
7.266
5.569
1.758
1.260
1.253
1.246
0.000
0.92
0.99
0.90
3.39
6.24
1.95
10.5 10.0 9.5 9.0 8.5 8.0 7.5 7.0 6.5 6.0 5.5 5.0 4.5 4.0 3.5 3.0 2.5 2.0 1.5 1.0 0.5 0.0
f1 (ppm)


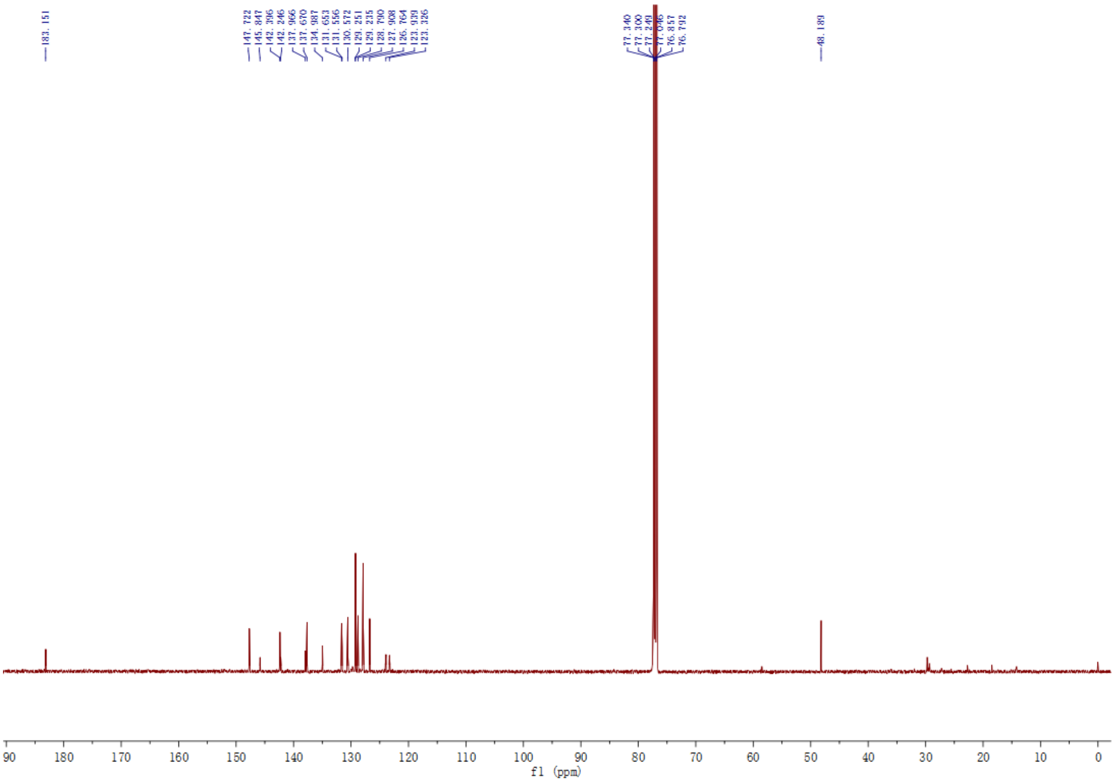

183.151
147.722
145.847
142.396
142.246
137.966
137.670
134.987
131.653
131.556
130.572
129.251
129.235
128.790
127.908
126.764
123.939
123.326
77.340
77.300
77.249
77.046
76.857
76.792
48.189
90 180 170 160 150 140 130 120 110 100 90 80 70 60 50 40 30 20 10 0
f1 (ppm)

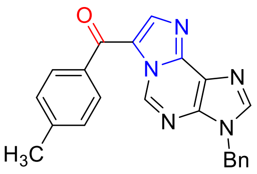
O
N
N
N
N
N
H3C
Bn

**2l**

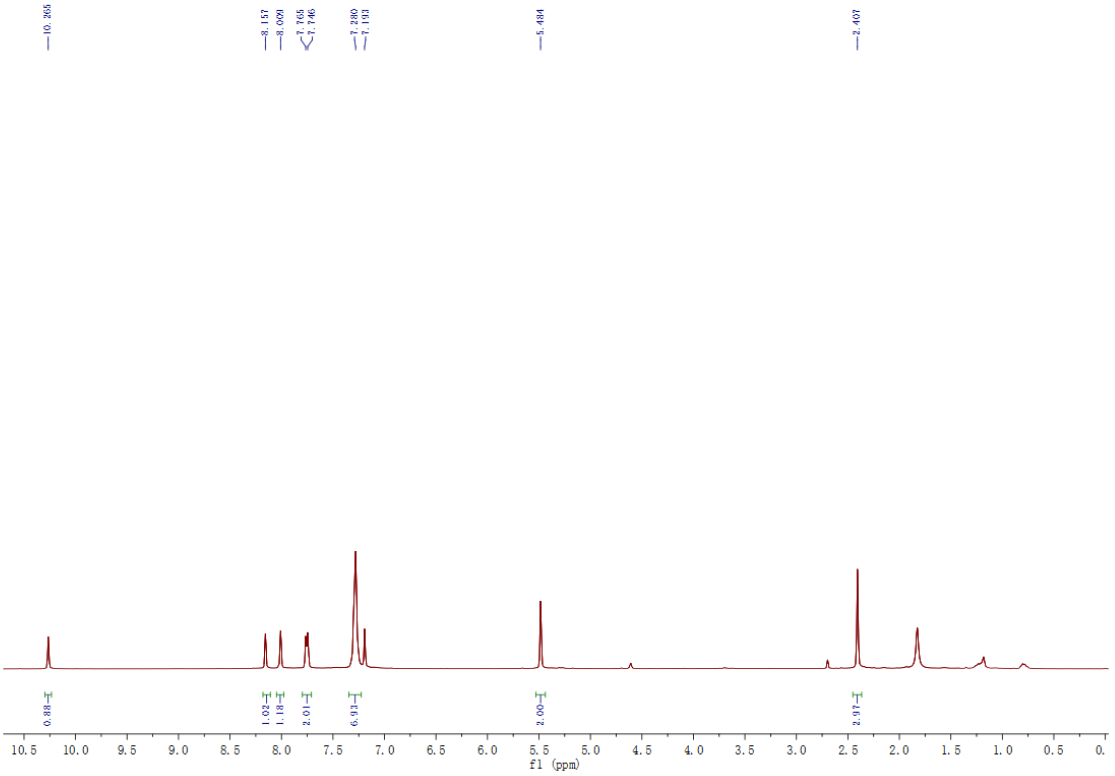
10.265
8.157
8.009
7.765
7.746
7.280
7.193
5.484
2.407
0.88
1.02
1.18
2.01
6.93
2.00
2.97
f1 (ppm)

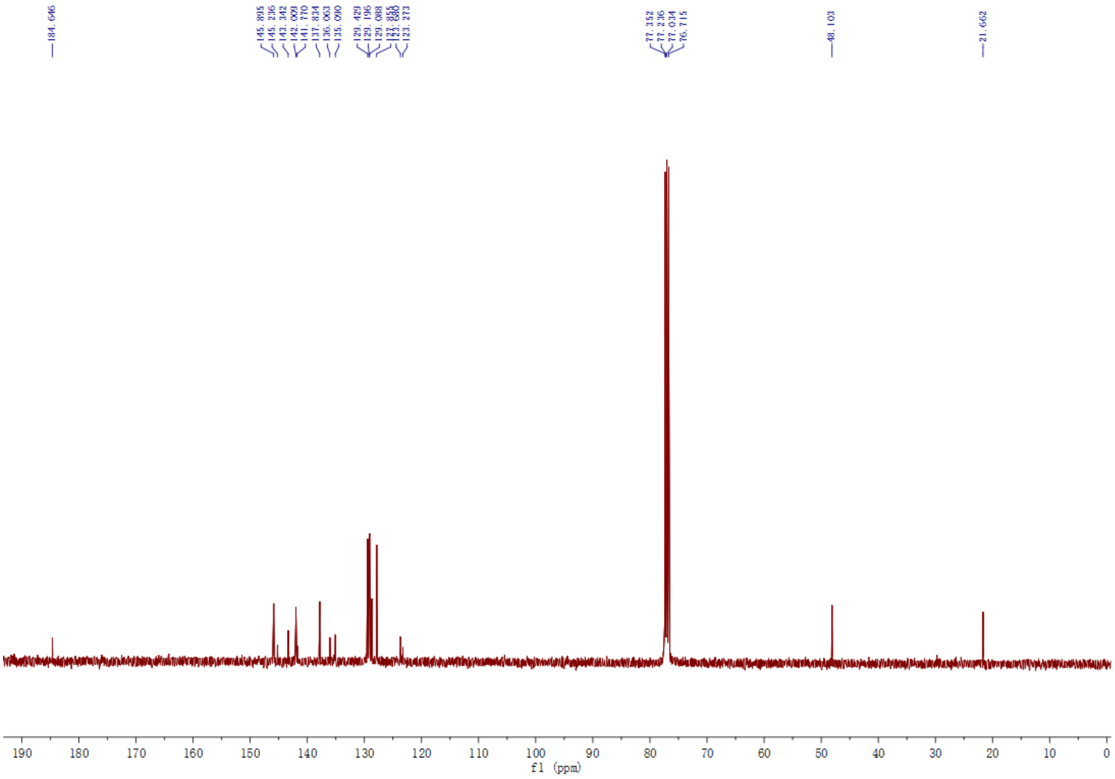
184.646
145.895
145.236
143.342
142.009
141.770
137.834
136.063
135.090
129.429
129.196
129.088
123.273
77.352
77.236
77.034
76.715
48.103
21.662
f1 (ppm)

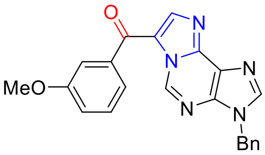


**2n**

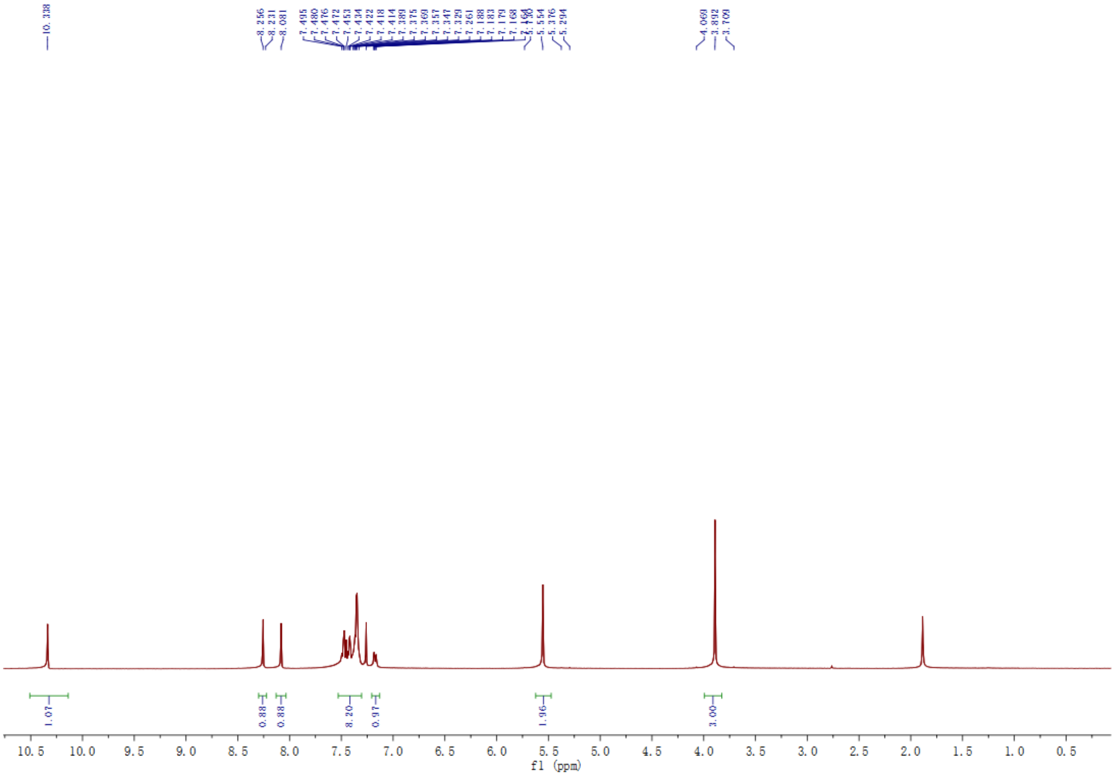


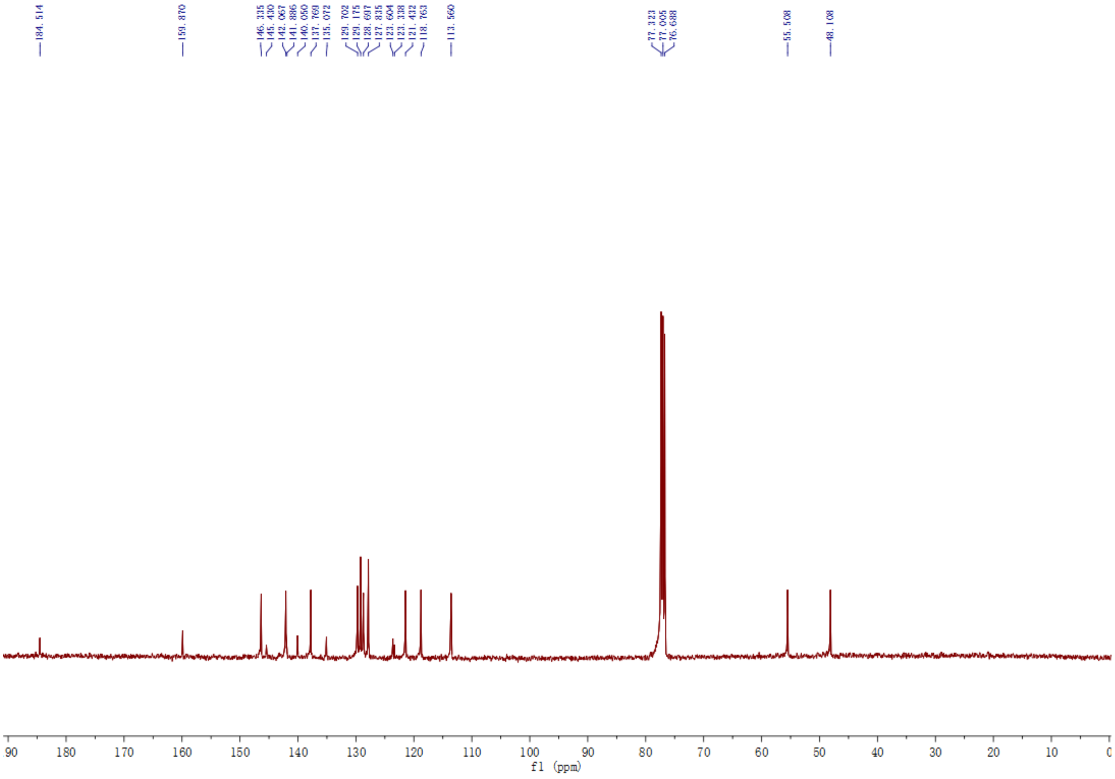

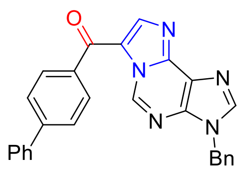
O
N
N
N
N
N
Ph
Bn

**2p**

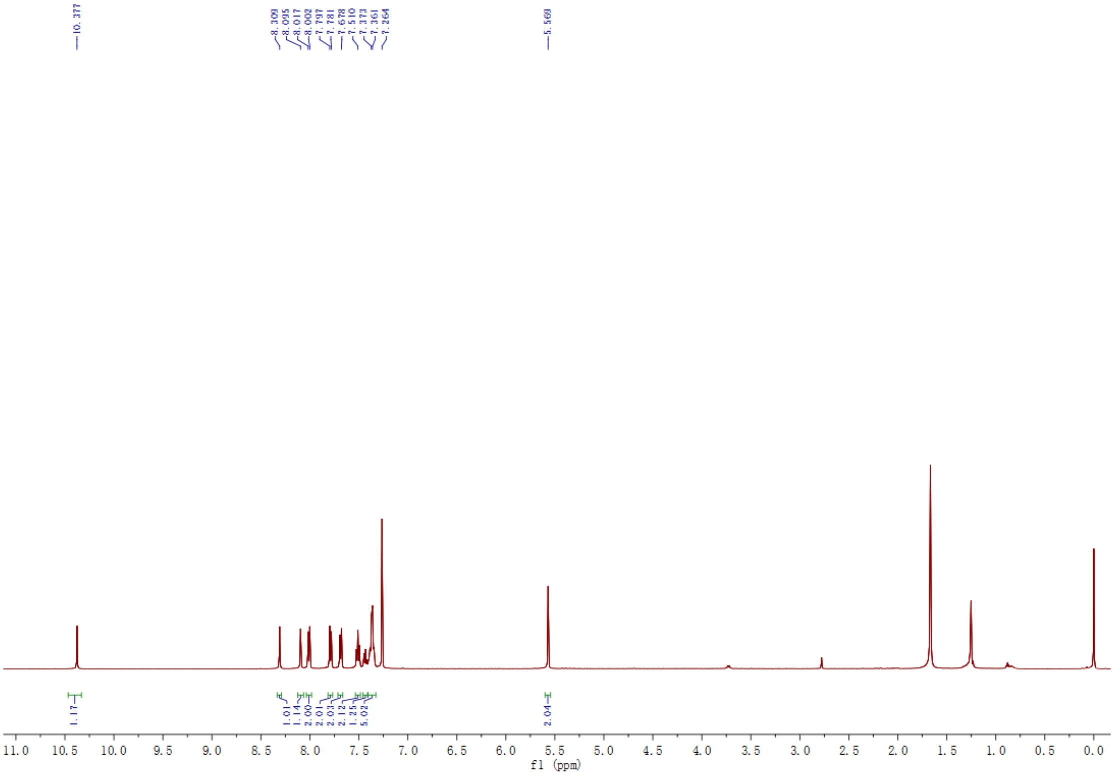
10.377
8.309
8.095
8.017
8.002
7.797
7.781
7.678
7.510
7.373
7.361
7.264
5.569
1.17
1.01
1.14
2.00
2.01
2.03
2.12
1.25
5.02
2.04
11.0
10.5
10.0
9.5
9.0
8.5
8.0
7.5
7.0
6.5
6.0
5.5
5.0
4.5
4.0
3.5
3.0
2.5
2.0
1.5
1.0
0.5
0.0
f1 (ppm)

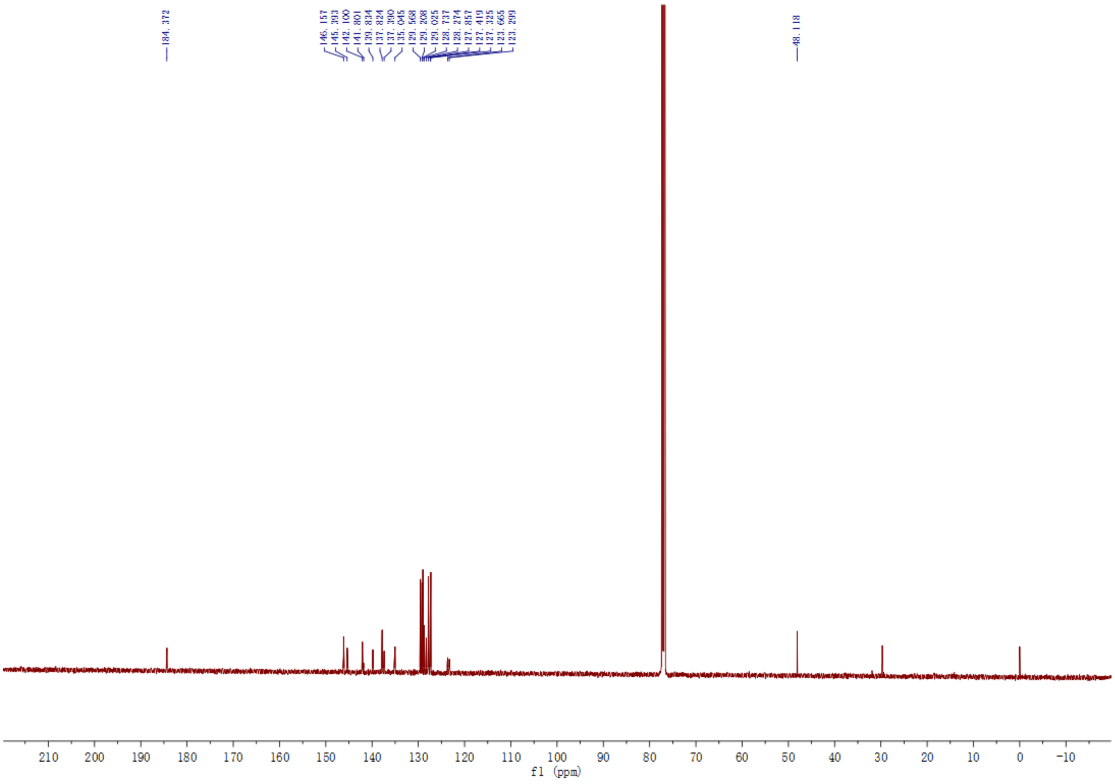
184.372
146.157
145.393
142.100
141.801
139.834
137.824
137.290
135.045
129.568
129.208
129.025
128.737
128.274
127.857
127.419
127.325
123.665
123.299
48.118
210
200
190
180
170
160
150
140
130
120
110
100
90
80
70
60
50
40
30
20
10
0
-10
f1 (ppm)

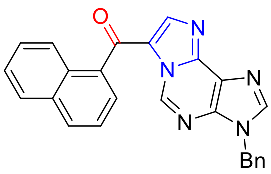
O
N
N
N
N
N
Bn

**2q**

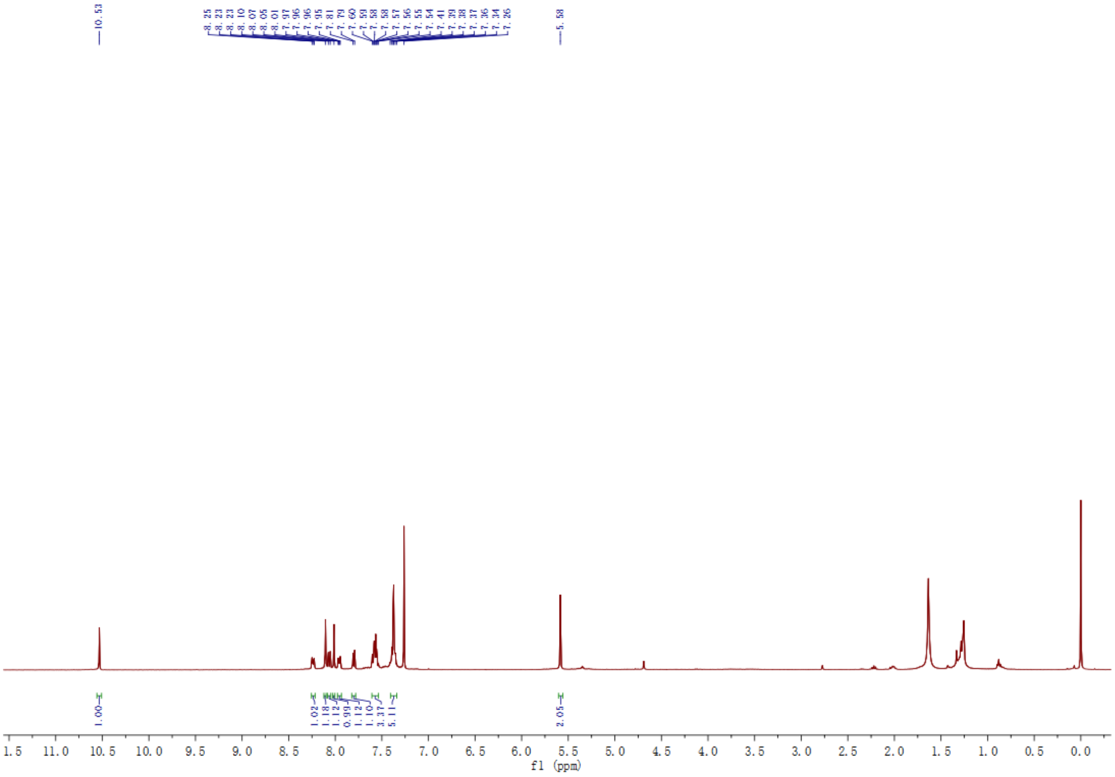
10.53
8.25
8.23
8.23
8.10
8.07
8.05
8.01
7.97
7.96
7.96
7.95
7.81
7.79
7.60
7.59
7.58
7.58
7.57
7.56
7.55
7.54
7.41
7.39
7.38
7.37
7.36
7.34
7.26
5.58
1.00
1.02
1.18
1.12
0.99
1.12
1.10
3.17
5.11
2.05
f1 (ppm)

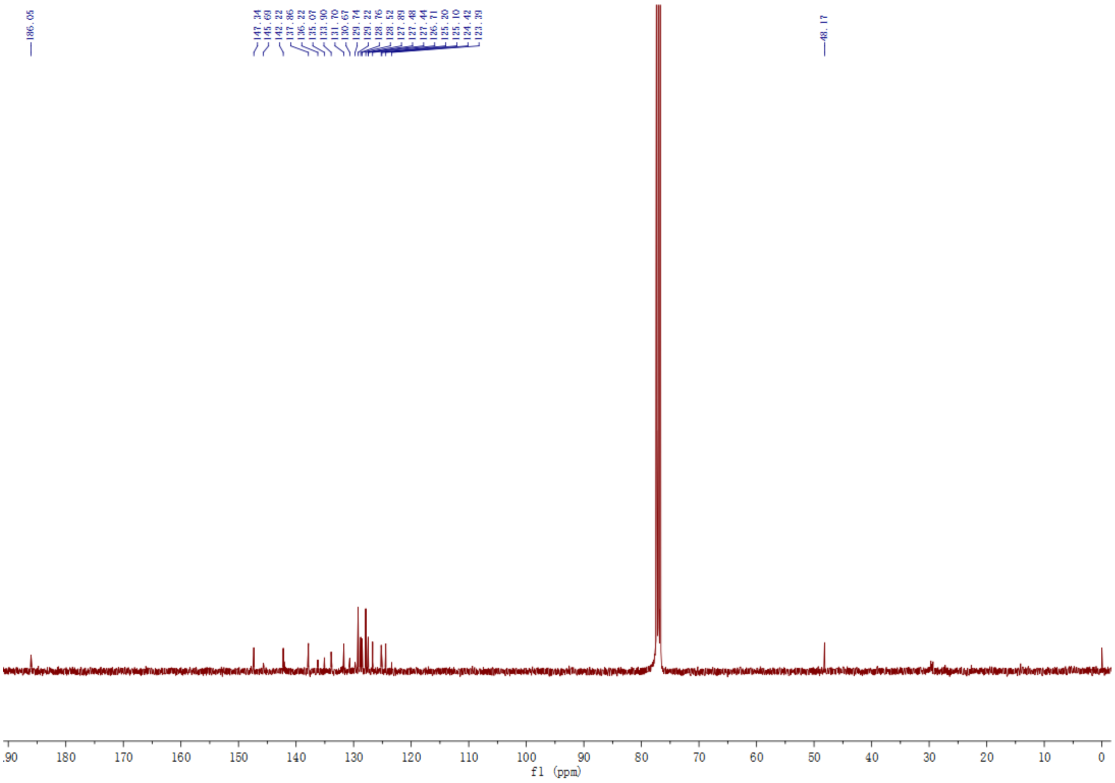
186.05
147.34
145.69
142.22
137.86
136.22
135.07
133.90
131.70
130.67
129.74
129.22
128.76
128.52
127.89
127.48
127.44
126.71
125.30
125.10
124.42
123.39
48.17
f1 (ppm)

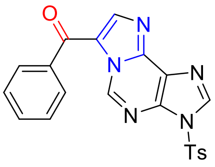
O
N
N
N
N
N
Ts

**2r**

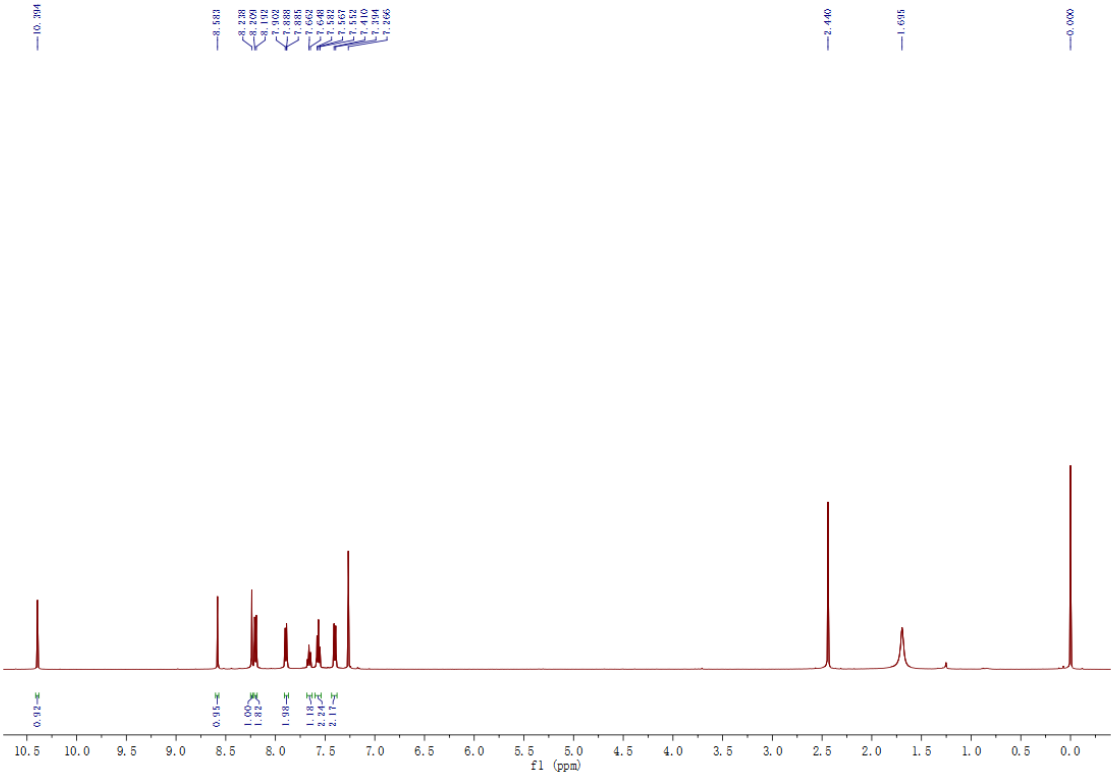
10.394
8.583
8.238
8.209
8.192
7.902
7.888
7.885
7.662
7.648
7.582
7.567
7.552
7.410
7.394
7.266
2.440
1.695
0.000
0.92
0.95
1.00
1.82
1.98
1.18
2.24
2.17
f1 (ppm)

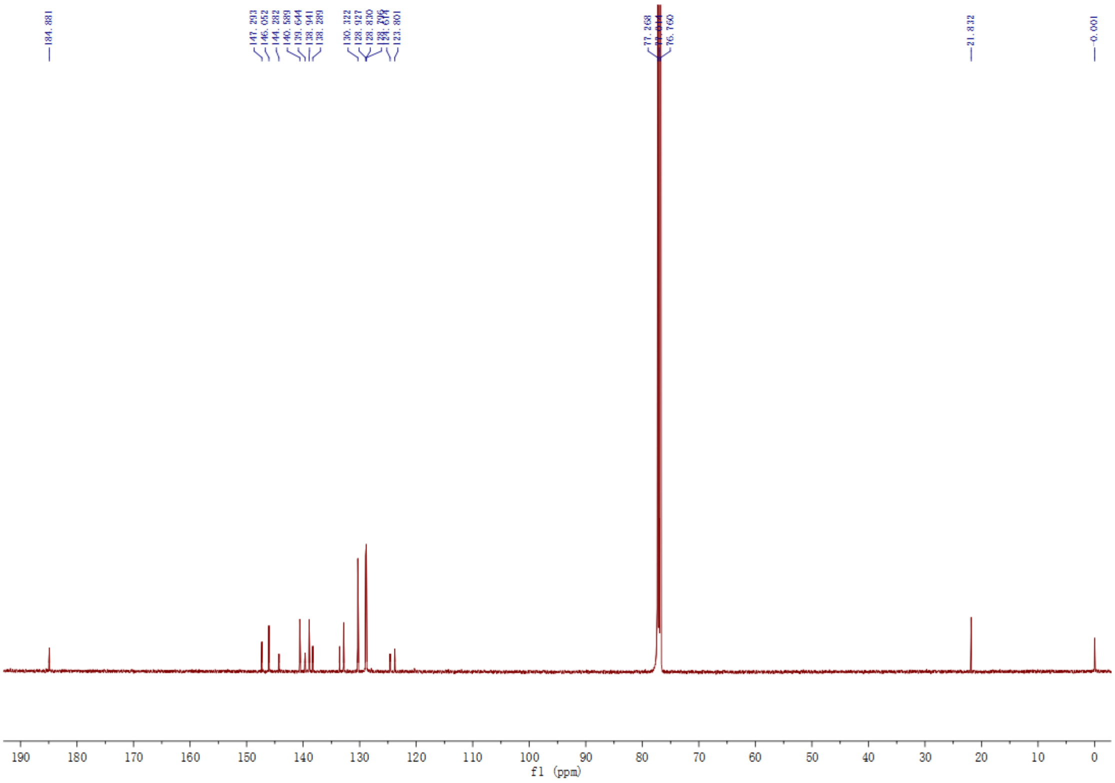
184.881
147.293
146.052
144.282
140.589
139.644
138.941
138.289
130.322
128.927
128.830
123.801
77.268
76.760
21.832
-0.001
f1 (ppm)

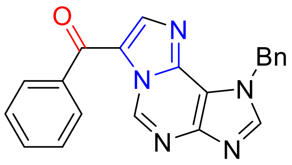
O
N
N
Bn
N
N
N

**2s**

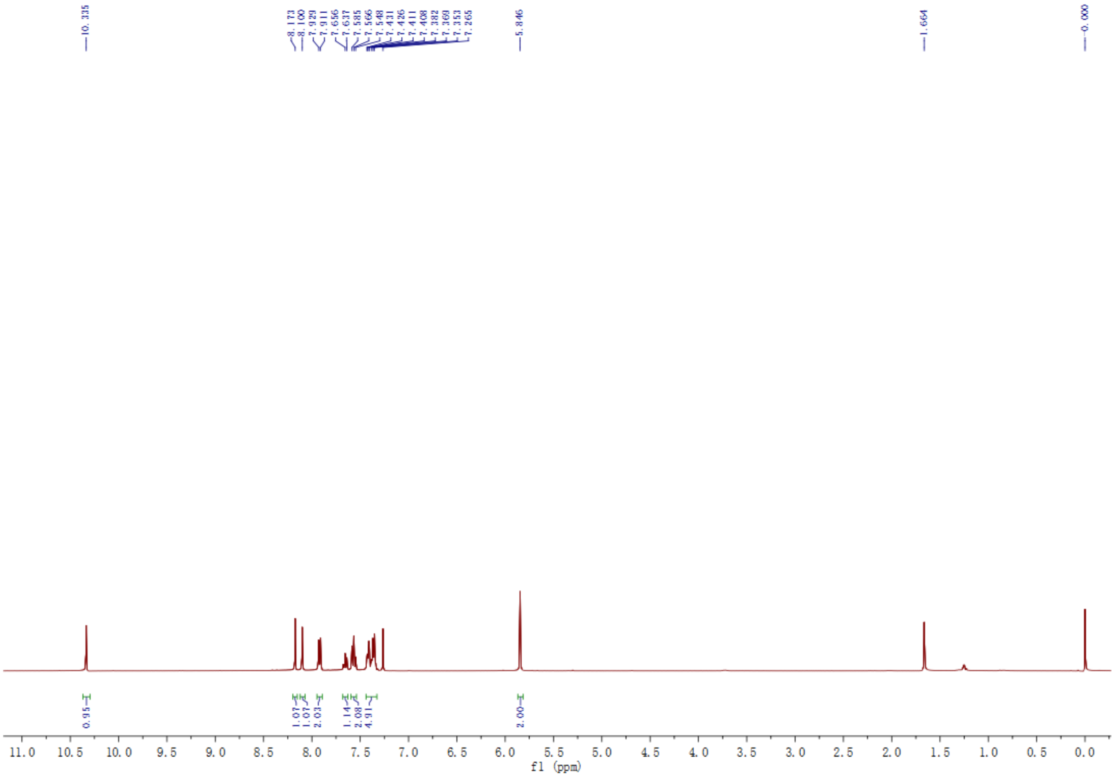
10.335
8.173
8.100
7.929
7.911
7.656
7.637
7.585
7.566
7.548
7.431
7.426
7.411
7.408
7.382
7.369
7.353
7.265
5.846
1.664
-0.000
0.95
1.07
1.07
2.03
1.14
2.08
4.91
2.00
11.0 10.5 10.0 9.5 9.0 8.5 8.0 7.5 7.0 6.5 6.0 5.5 5.0 4.5 4.0 3.5 3.0 2.5 2.0 1.5 1.0 0.5 0.0
f1 (ppm)

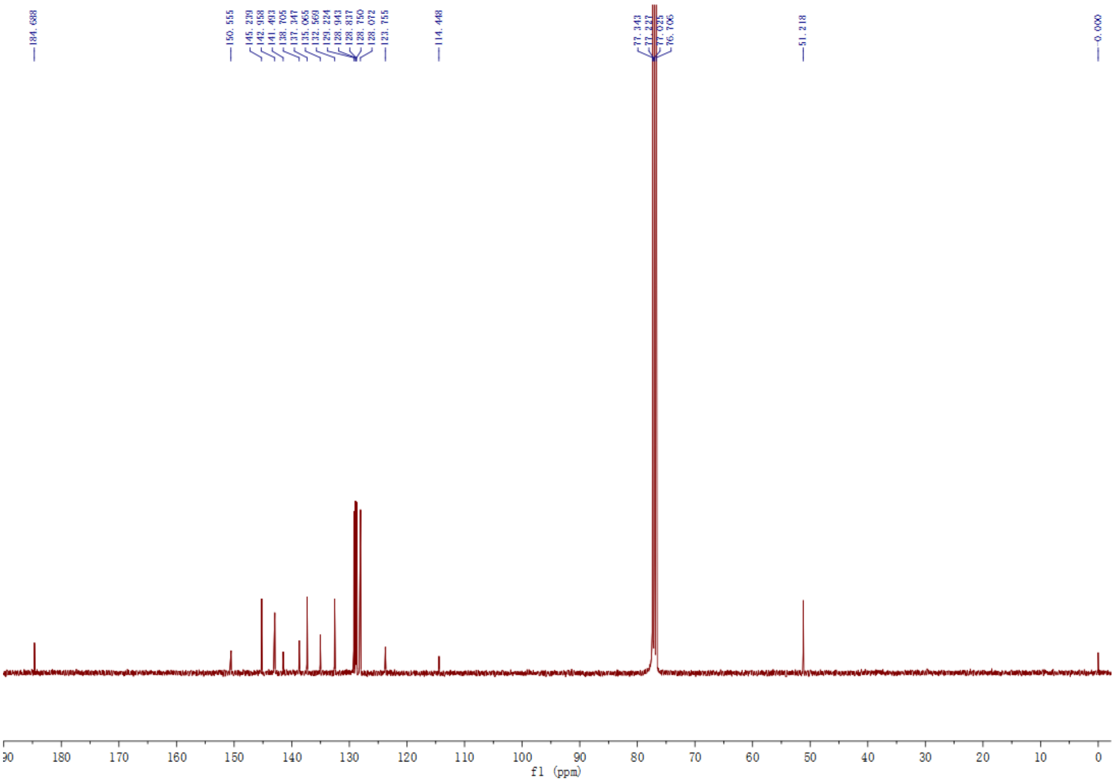
184.688
150.555
145.239
142.958
141.493
138.705
137.347
135.065
132.569
129.224
128.943
128.837
128.750
128.072
123.755
114.448
77.343
77.227
77.025
76.706
51.218
-0.000
90 180 170 160 150 140 130 120 110 100 90 80 70 60 50 40 30 20 10 0
f1 (ppm)

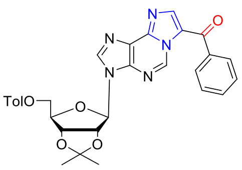
N
N
N
N
N
O
TolO
O
O
O

**2t**

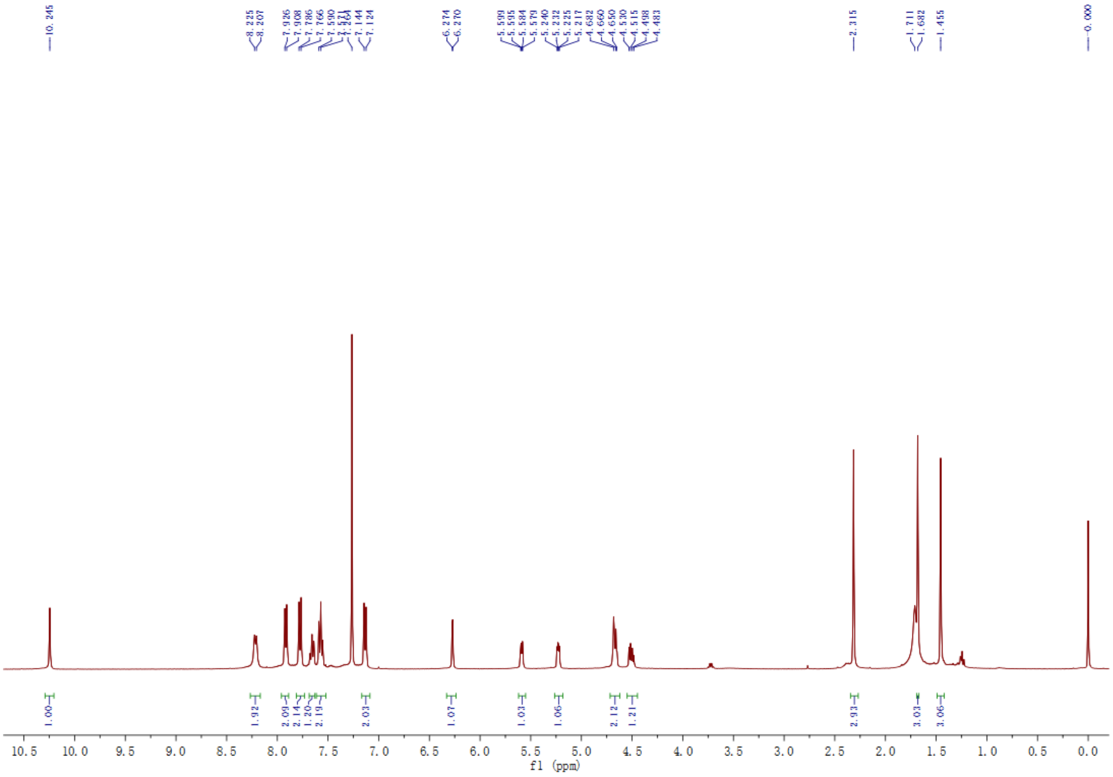
10.245
8.225
8.207
7.926
7.908
7.786
7.766
7.590
7.144
7.124
6.274
6.270
5.599
5.595
5.584
5.579
5.240
5.232
5.225
5.217
4.682
4.660
4.650
4.530
4.515
4.498
4.483
2.315
1.711
1.682
1.455
0.000
1.00
1.92
2.09
2.14
1.20
2.19
2.03
1.07
1.03
1.06
2.12
1.21
2.93
3.03
3.06
10.5
10.0
9.5
9.0
8.5
8.0
7.5
7.0
6.5
6.0
5.5
5.0
4.5
4.0
3.5
3.0
2.5
2.0
1.5
1.0
0.5
0.0
f1 (ppm)

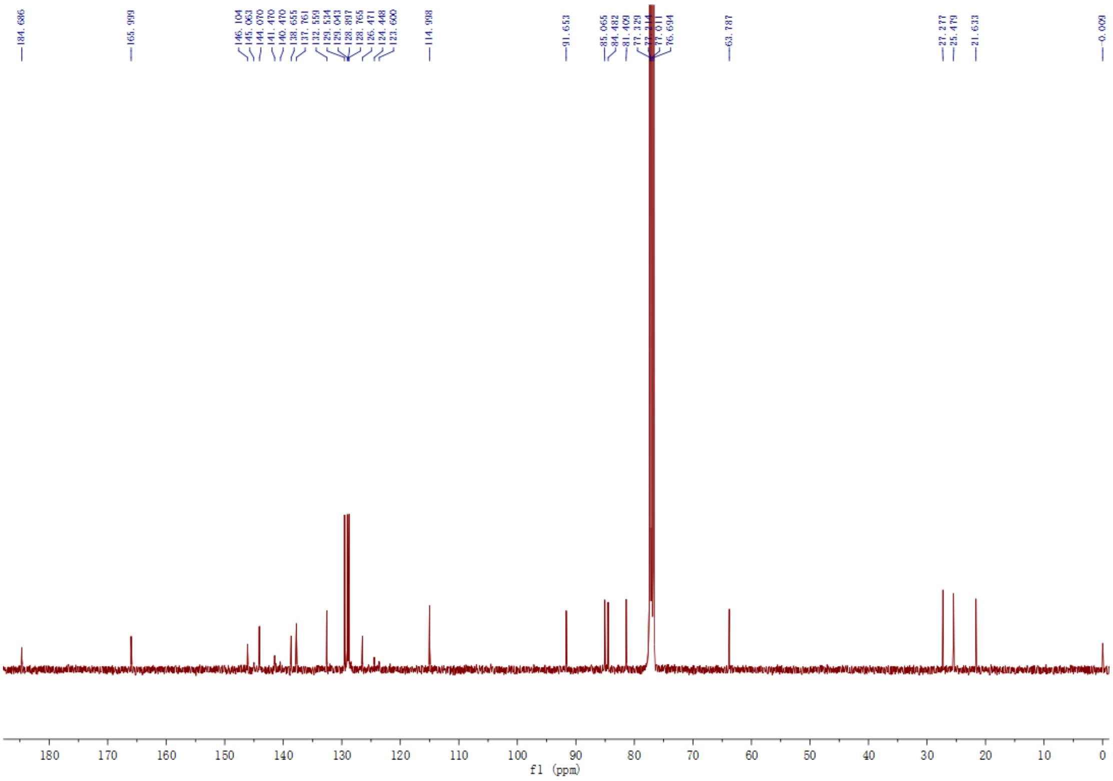
184.686
165.999
146.104
145.063
144.070
141.470
140.470
138.655
137.761
132.559
129.534
129.043
128.897
128.765
126.471
124.448
123.600
114.998
91.653
85.065
84.482
81.409
77.329
77.011
76.694
63.787
27.277
25.479
21.633
-0.009
180
170
160
150
140
130
120
110
100
90
80
70
60
50
40
30
20
10
0
f1 (ppm)

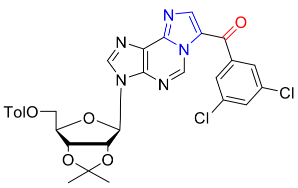


**2u**

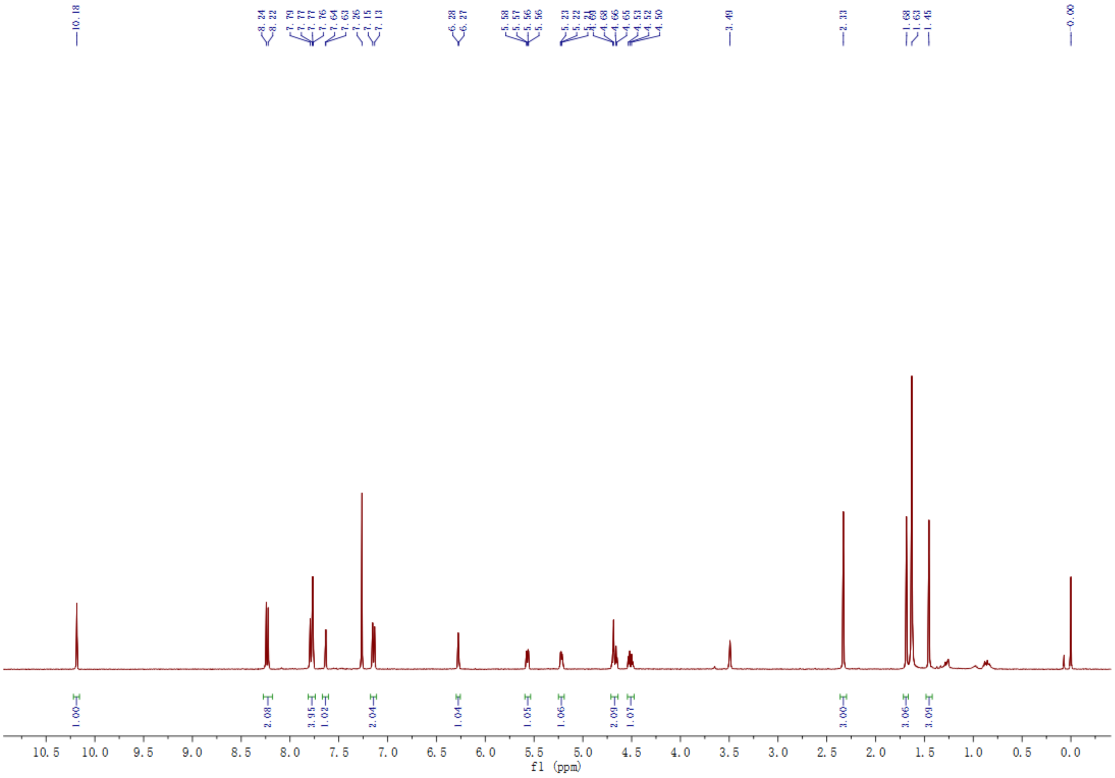


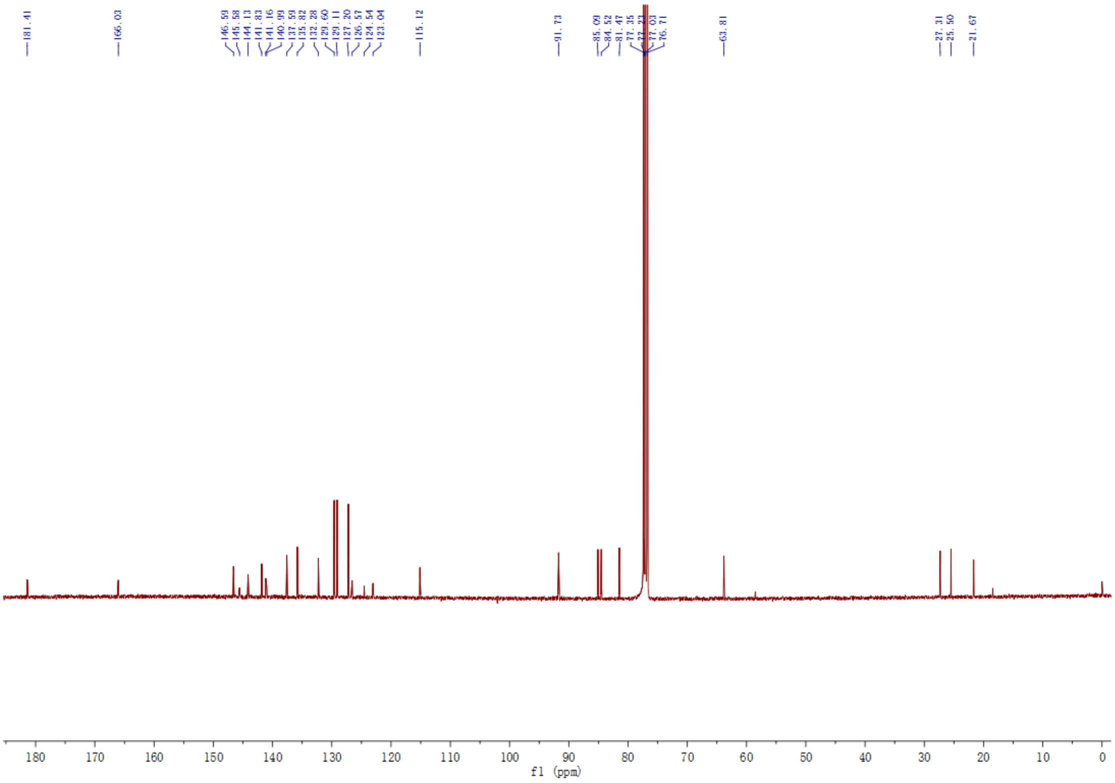

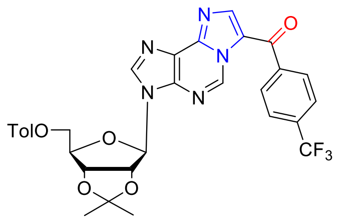
TolO
$CF_3$

**2v**

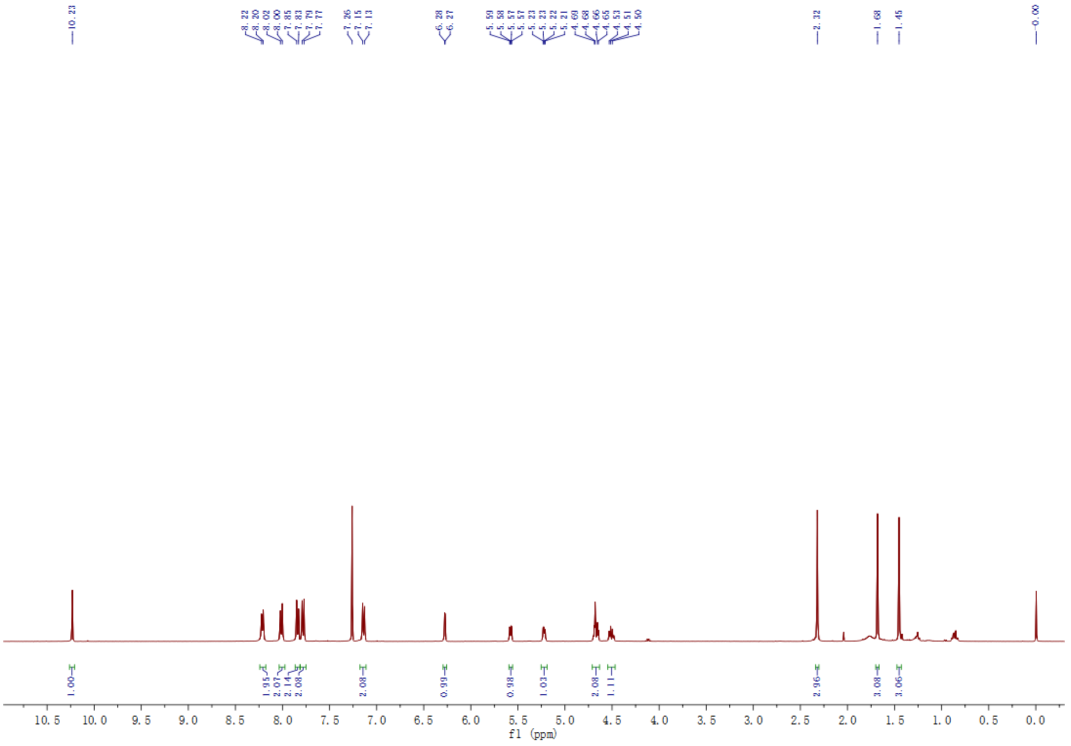
10.23
8.22 8.20 8.02 8.00 7.85 7.83 7.79 7.77
7.26 7.15 7.13
6.28 6.27
5.59 5.58 5.57 5.57 5.23 5.23 5.22 5.21 4.69 4.68 4.66 4.65 4.53 4.51 4.50
2.32
1.68
1.45
0.00
1.00 1.95 2.07 2.14 2.08 2.08 0.99 0.98 1.03 2.08 1.11 2.96 3.08 3.06
f1 (ppm)

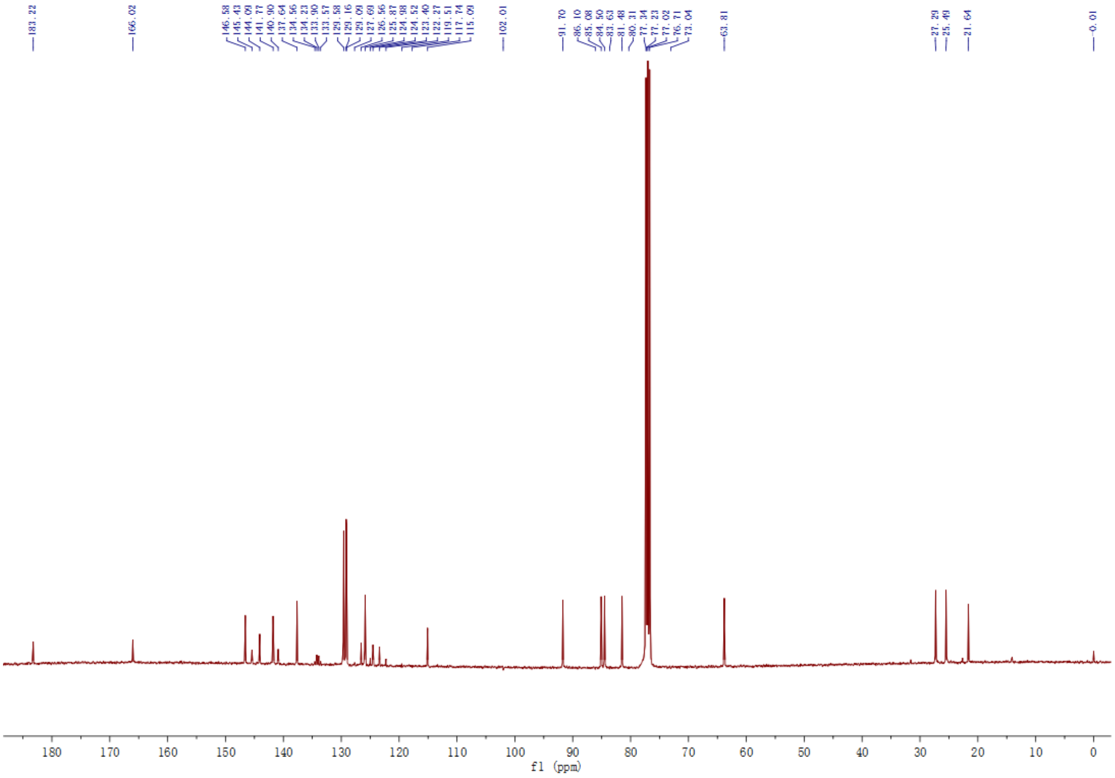
183.22
166.02
146.58 145.43 144.09 141.77 140.90 137.64 134.56 134.23 133.90 133.57 129.58 129.16 129.09 127.69 126.56 125.87 124.98 124.52 123.40 122.27 119.51 117.74 115.09
102.01
91.70 85.10 85.08 84.50 83.63 81.48 80.31 77.34 77.23 77.02 76.71 73.04
63.81
27.29 25.49 21.64
0.01
f1 (ppm)

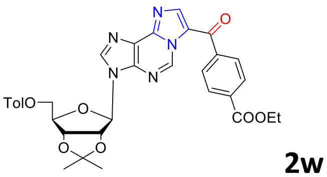

N
N
N
N
O
TolO
O
O
O
COOEt


**2w**

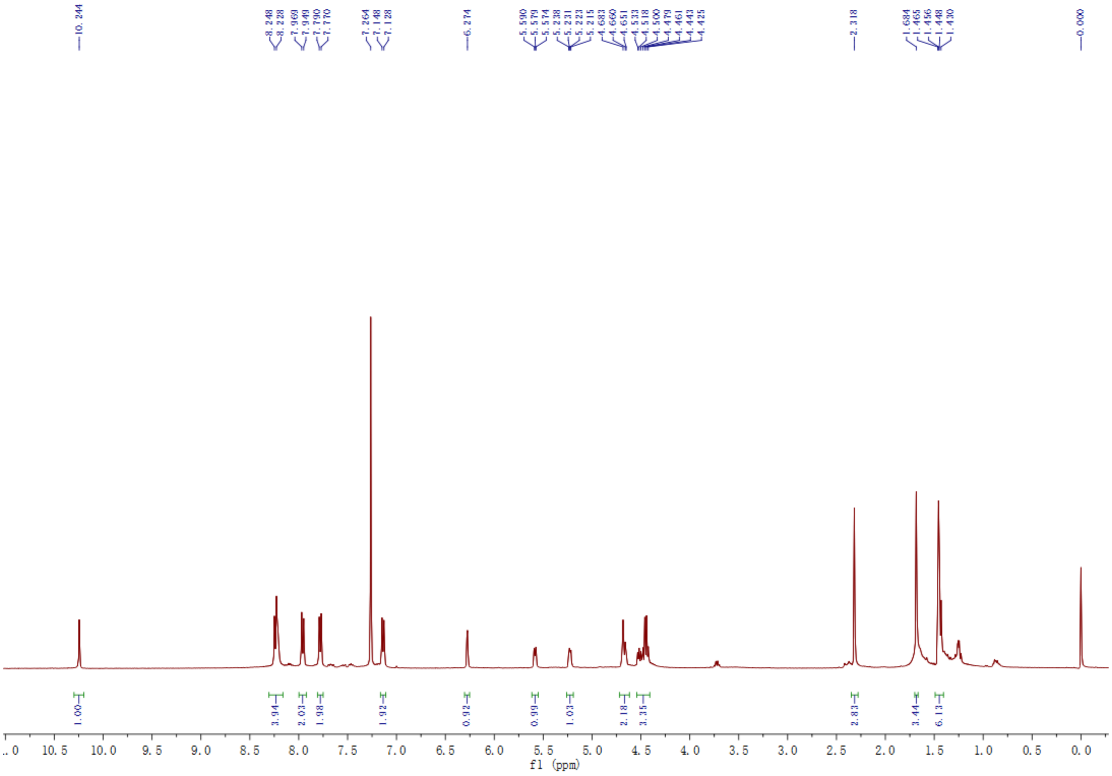

10.244
8.248
8.228
7.969
7.949
7.790
7.770
7.264
7.148
7.128
6.274
5.590
5.579
5.574
5.238
5.231
5.223
5.215
4.683
4.660
4.651
4.533
4.518
4.500
4.479
4.461
4.443
4.425
2.318
1.684
1.465
1.456
1.448
1.430
0.000
1.00
3.94
2.03
1.98
1.92
0.92
0.99
1.03
2.18
3.35
2.83
3.44
6.13
f1 (ppm)


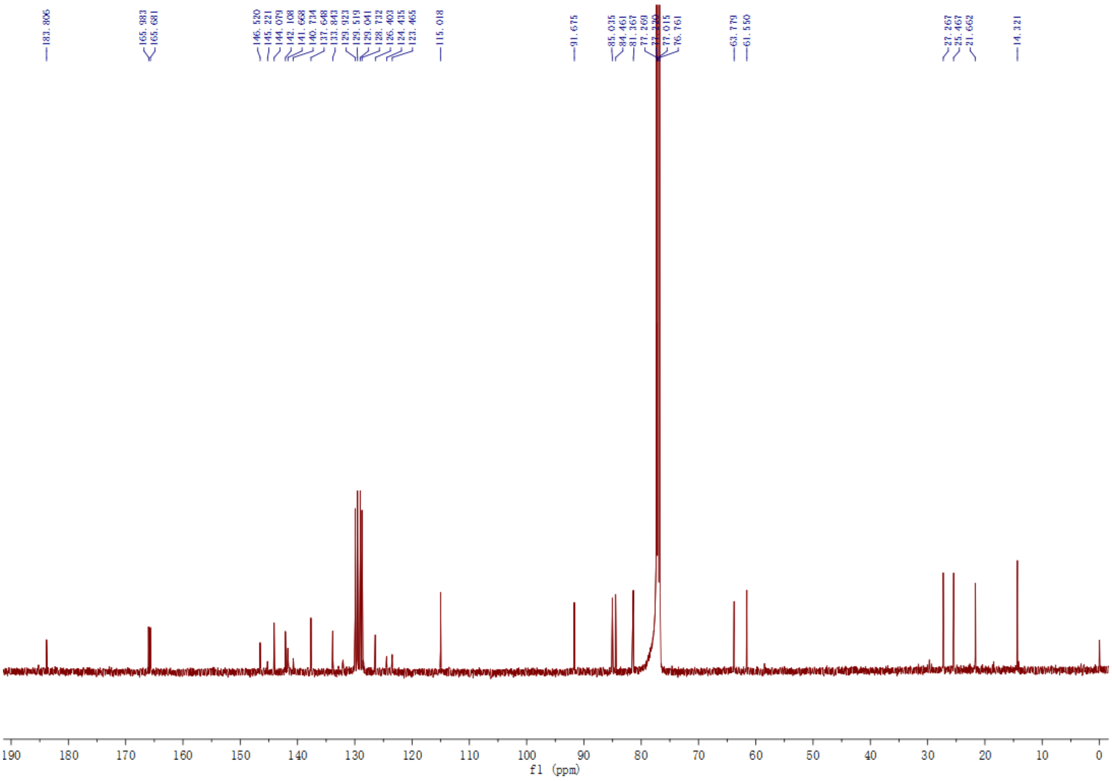

183.806
165.983
165.681
146.520
145.221
144.079
142.108
141.668
140.734
137.648
133.843
129.923
129.519
129.041
128.732
126.403
124.435
123.465
115.018
91.675
85.035
84.461
81.367
77.269
77.015
76.761
63.779
61.550
27.267
25.467
21.662
14.321
f1 (ppm)
